# Evaluating MR enterography and intestinal ultrasound for ileal Crohn’s disease activity: a multicentre agreement study

**DOI:** 10.1007/s44501-026-00002-8

**Published:** 2026-09-11

**Authors:** Maira Hameed, Busola Adebusoye, Sue Mallett, Gauraang Bhatnagar, Anthony Higginson, Steve Halligan, Stuart A. Taylor

**Affiliations:** 1 University College London Hospitals, London, UK; 2 Centre for Medical Imaging, University College London, London, UK; 3 Frimley Health NHS Trust, Frimley, UK; 4 Portsmouth Hospitals University NHS Trust, Portsmouth, UK; 5 University College London, London, UK; 6 St James’s University Hospital, Leeds Teaching Hospitals NHS Trust, Leeds, UK; 7 Norwich and Norfolk University Hospitals NHS Foundation Trust, Norwich, UK; 8 Oxford University Hospitals NHS Trust, Oxford, UK; 9 Royal Free London NHS Foundation Trust, London, UK; 10 St Mark’s Hospital, LNWUH NHS Trust, Harrow, UK; 11 St George’s University Hospitals NHS Foundation Trust, London, UK; 12 Ninewells Hospital, Dundee, UK; 13 Institute of Applied Health Research, National Institute of Health and Research Birmingham Biomedical Research Centre, College of Medical and Dental Sciences, University of Birmingham, Birmingham, UK; 14 Department of Psychological Sciences, Birkbeck University of London, London, UK

**Keywords:** Magnetic resonance imaging, Ultrasonography, Crohn’s disease

## Abstract

**Objectives:**

We aimed to establish the level of agreement between signs of Crohn’s disease activity common to MR Enterography (MRE) and Intestinal Ultrasound (IUS).

**Materials and methods:**

Retrospective review of the METRIC multicentre prospective cohort trial (ISRCTN03982913) of newly diagnosed or suspected relapsed Crohn’s disease undergoing contemporaneous MRE and IUS. Intermodality agreement for prespecified disease variables (10 MRE, 14 IUS) from the terminal ileum was assessed using Bland–Altman plots and percentage agreement against a multidisciplinary consensus panel reference standard. Imputation was used to account for disease not identified on either modality.

**Results:**

284 patients were included, median age 36 years (interquartile range, IQR: 17–68), 154/284 (54%) female. In the full cohort, intermodality 95% limits of agreement for terminal ileal mural thickness and disease length were −4.8 to 6.7 mm and −22.8 to 23.6 cm, respectively, and in the 158 patients with disease identified on both modalities, −4.0 to 6.3 mm and −27.5 to 29.1 cm, respectively. Increased mural T2 signal demonstrated the highest concordance with IUS submucosal layer thickening (91%, 141/155). Increased perimural T2 signal showed limited agreement with various IUS mesenteric observations (32–48%). There was 95% (147/155) agreement for global judgement of segmental disease severity and 74% (116/157) for obstruction.

**Conclusion:**

While intermodality agreement for disease severity and obstruction is reasonable in patients with disease identified on both modalities, clinically important disagreement for mural thickness and disease length is frequent. Findings suggest caution in comparing individual parameters in isolation with potentially clinically significant intermodality disagreement.

**Key Points:**

**Question** *MR Enterography and Intestinal Ultrasound are used interchangeably in Crohn’s disease, but comparison between modalities is challenging.*

**Findings** *We found reasonable intermodality agreement for global segmental disease activity but poor agreement for mural thickness, disease length, and most modality-specific activity parameters.*

**Relevance statement** *This limited agreement is clinically significant with the potential to impact management decisions. Findings suggest caution in comparing individual disease parameters in isolation between MR Enterography and Intestinal Ultrasound. Instead, a holistic judgement of activity is more reliable.*

**Graphical Abstract:**

**Figure:**
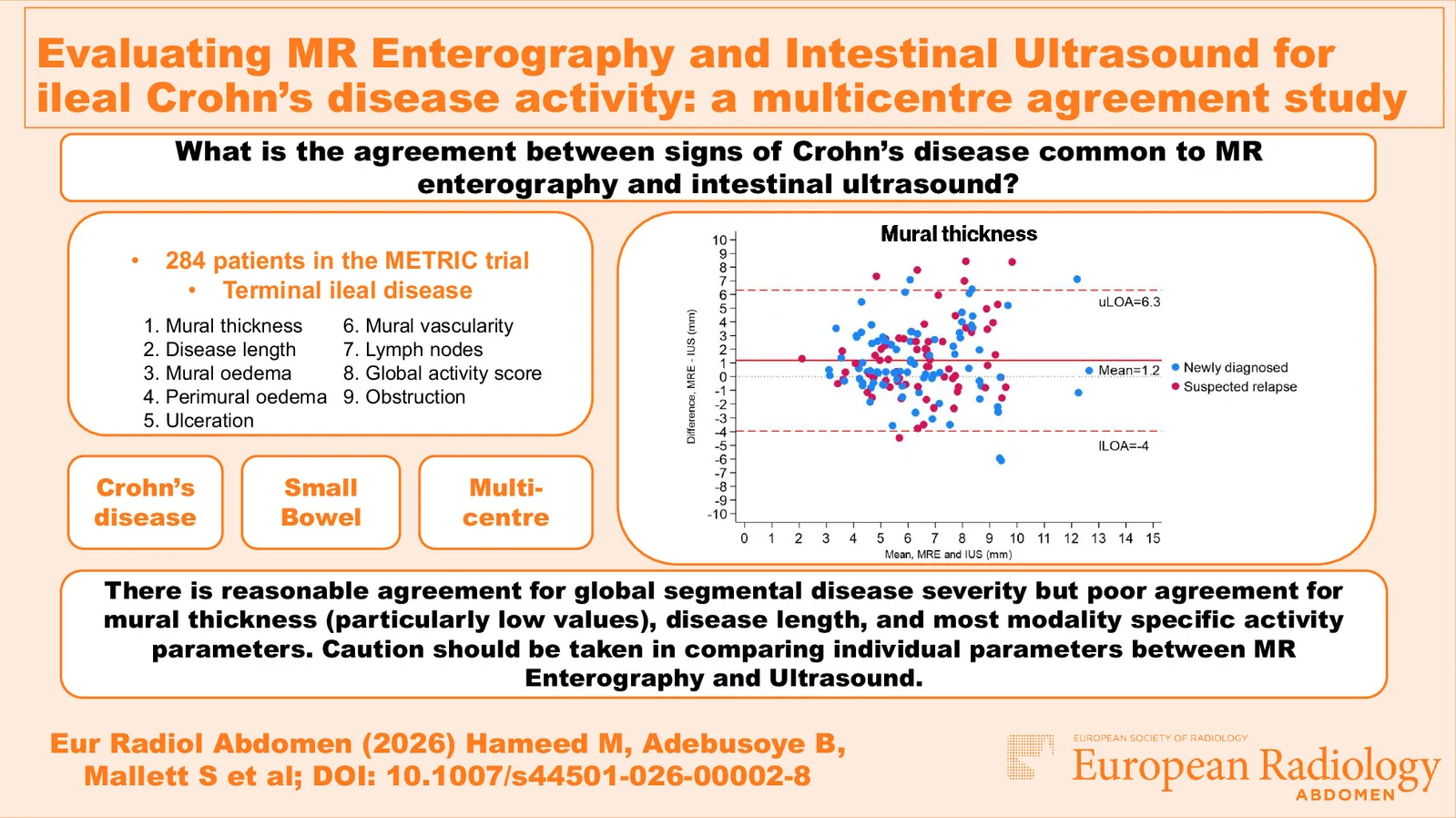

## Introduction

Cross-sectional imaging, in particular, MR Enterography (MRE) and Intestinal Ultrasound (IUS), play a key role in small bowel Crohn’s disease (CD) management [1, 2]. Both assess inflammatory burden accurately and facilitate objective treatment response assessment, essential to guide appropriate therapy and avoid irreversible bowel damage [3, 4].

Imaging signs of active disease have been derived using endoscopic, biochemical, and histopathological reference standards, and include bowel wall thickening, mural and perimural oedema, ulceration, and hypervascularity/neoangiogenesis [1, 5–8]. These parameters are included in multiple MRE and IUS disease activity scores [5, 7, 9, 10]. Some parameters, such as wall thickness and disease length, are common to both MRE and IUS, but others, for example, mural oedema, are exclusive to each modality, reflecting their differing physical principles [1, 5].

The prospective, multicentre METRIC trial found that both MRE and IUS exhibited good sensitivity and specificity for diagnosis and evaluating disease relapse (96% and 83% for MRE, and 90% and 77% for IUS for active disease presence), but did not specifically address the intermodality agreement of potentially equivalent signs of disease activity [11]. In current clinical practice, the choice of imaging modality for follow-up is discretionary and influenced by clinician and patient preference, and available imaging resources and expertise. It is therefore not uncommon for different modalities to be used before and after treatment, potentially confounding judgement of response. Data on the interchangeability of MRE and IUS for disease activity are sparse, and it is unclear how response is best assessed. Prior studies are typically small (< 30 patients), single centre, and/or limited to mural thickness and disease length agreement [12–18]. Indeed, even the simplest parameter of bowel wall thickness may vary between modalities depending on bowel distension and image resolution [8]. Addressing this uncertainty has potential implications for optimising imaging pathways, as well as training and service development.

Using METRIC data, we aimed to establish agreement between MRE and IUS for (1) validated signs of disease activity, (2) other commonly assessed parameters, including disease length and the presence of obstruction.

## Methods

### Study summary

Retrospective review of the METRIC multicentre prospective cohort trial (ISRCTN03982913) of 284 patients, aged 16 years and older, with newly diagnosed or suspected relapsed CD [11, 19]. The primary aim was to compare the per-patient sensitivity of MRE and IUS for small bowel disease extent. The METRIC study received research ethics committee approval in September 2013 (13/SC/0394), and informed, written consent was obtained, including patient consent for use of their data for other research. Patients were recruited from 8 UK teaching and general hospitals, between December 2013 and September 2016 inclusive, and underwent both MRE and IUS within 4 weeks. Researchers prospectively and independently recorded disease presence and activity for each modality using study report forms. Interpretation was blind to the opposing modality and the reference standard. Parameters related to disease activity were pre-defined by the trial protocol. Full inclusion and exclusion criteria, and trial interventions, including imaging protocols, are detailed in the main trial protocol [11, 19]. Patients in the hydrosonography sub-study were not included.

### MRE and IUS parameters and scoring

Overall, 28 study practitioners (27 gastrointestinal radiologists with 9 years median experience [IQR: 5–11], 1 sonographer with 20 years of experience) interpreted MRE and IUS. If disease was deemed present, for each affected bowel segment, parameters of disease activity for both modalities were recorded (Table 1); examples are presented in Appendices 1 and 2. Disease length and evidence of obstruction (if any) were also documented, along with a global segmental disease severity classification (“none”, “mild” or “advanced”) using a priori definitions. Obstruction was defined as upstream bowel dilation compared to normal bowel calibre. Mural thickness was defined as the maximal measurement from the mucosal to serosal layer. We included only terminal ileal cases in this present analysis, as most cases were in this location (217/284), and the assumption was that segmental disease matching between modalities would be most accurate here and more likely to have an ileocolonoscopic reference standard. If terminal ileal disease was contiguous for ≥ 10 cm, it was defined as ‘terminal ileum’ (Appendix 2). As previously reported, practitioners graded the visualisation of the terminal ileum as good, moderate or poor [20].

**Table 1:** Individual MRE and IUS parameters mapped between modalities

MRE parameter	Parameter score coding as active/inactive, MRE	IUS parameter	Parameter score coding as active/inactive, IUS
**1. Mural thickness** (mm)	n/a	**1. Mural thickness** (mm)	n/a
2. Mural T2 signal* 0: Equivalent to normal bowel wall T2 signal 1: Minor increase (dark grey) 2: Moderate increase (light grey) 3: Marked increase (approaching luminal content)	Active: 1, 2, 3 Inactive: 0	**2a. Submucosal layer** 0: Normal 1: Thickened **2b. Submucosal layer echogenicity** 0: Normal 1: Reduced 2: Uniform increased 3: Increased with bands **2c. Submucosal layer clarity** 0: Well-defined 1: Ill-defined **2d. Mucosal layer** 0: Well-defined 1: Isolated thickened 2: Thickened + submucosal thickening	Active: 1 Inactive: 0 Active: 1, 3^#^ Inactive: 0, 2, 3^#^ Active: 1 Inactive: 0 Active: 1, 2^#^ Inactive: 0, 2^#^
3. Perimural T2 signal* 0: Equivalent to normal mesentery 1: Increased but no fluid 2: Small fluid rim (< 2 mm) 3: Large fluid rim (> 2 mm)	Active: 1, 2, 3 Inactive: 0	**3a. Mesenteric fat echogenicity** 0: Normal 1: Focal hyperechoic 2: Focal hyperechoic + fat wrap 3: Stratified heterogeneous + fat wrap 4: Uniform hypoechoic **3b. Anti-mesenteric border** 0: Well-defined 1: Ill-defined **3c. Mesenteric border definition** 0: Well-defined 1: Generally ill-defined 2: Focally ill-defined	Active: 1, 2 Inactive: 0, 3, 4 Active: 1 Inactive: 0 Active: 1, 2 Inactive: 0
**4a. Mural enhancement** 0: Equivalent to normal bowel wall 1: Minor increase but significantly less than vessels 2: Moderate increase but somewhat less than vessels 3: Marked increase, approaching that of vessels **4b. Mural enhancement pattern** 0: n/a 1: Homogeneous 2: Mucosal 3: Layered	Active: 1, 2, 3 Inactive: 0 Active: 1, 2, 3 Inactive: 0	**4. Colour Doppler signal** 0: Normal 1: Increased focal (< 50% axial circumference) 2: Increased generalised	Active: 1, 2 Inactive: 0
**5. Ulceration** 0: None 1: Superficial (< 50% wall thickness) 2: Deep	Active: 1, 2 Inactive: 0	**5. Ulceration** 0–2 as with MRE	Active: 1, 2 Inactive: 0
**6. Lymph nodes** 0: Absent 1: Cluster < 1 cm 2: 1–2 nodes > 1 cm 3: 3 nodes > 1 cm	Active: 1, 2, 3 Inactive: 0	**6. Lymph nodes** 0–3 as with MRE	Active: 1, 2, 3 Inactive: 0
**7.** Segmental disease severity score** (0–2)	Active: 1, 2 Inactive: 0	**7.** Segmental disease severity score** (0–2)	Active: 1, 2 Inactive: 0
**8. Length of disease** (cm)	n/a	**8. Length of disease** (cm)	n/a
**9. Functional obstruction** 0: None 1: Upstream dilation	n/a	**9. Functional obstruction** 0–1 as with MRE	n/a

The range of possible scores for each parameter is given in brackets and grouped as denoting active or inactive disease, where relevant

Functional obstruction was defined as upstream bowel dilation relative to normal bowel calibre

*MRE* MR Enterography, *IUS* Intestinal Ultrasound, *mm* millimetres

* To denote oedema, on fat-saturated images

** 0: None; 1: Superficial ulcers and/or mild mural thickening and/or mild increased enhancement; 2: Advanced transmural disease and/or fistula and/or stricture and/or mural oedema or significant mural thickening

^#^ Active disease if with a colour Doppler score of 1 or 2, inactive if a colour Doppler score of 0

To facilitate direct comparison between modalities, individual activity parameters on MRE (*n* = 10) were mapped to at least one corresponding parameter on IUS (*n* = 14). Some parameters were directly comparable, for example, bowel wall thickness, but others had multiple potential equivalents on the opposing modality (Table 1). Accordingly, these parameters were grouped and coded as denoting active or inactive disease based on the available literature and opinion of the study team (Table 1, Appendices 1 and 2).

### Reference standard to define active disease

The reference standard for disease presence and activity in the METRIC trial was used for the current study [11, 20]. In brief, a multidisciplinary consensus panel, including at least one gastroenterologist and one radiologist from the recruitment site and one external radiologist, compiled a reference standard to define disease presence and activity. This was based on a review of all available clinical and imaging data up to 6 months after recruitment, including colonoscopy and histology when available.

### Statistical analysis summary

Intermodality agreement between individual parameters was assessed (Table 1), along with intermodality agreement for mural thickness (millimetres), disease length (centimetres), and obstruction.

Initial analysis included all patients (full cohort data) recruited to METRIC, including patients in whom one or both modalities had not visualised the disease (incorporating all perceptual and technical errors), meaning detailed activity parameters had not been recorded. The METRIC trial was powered to detect a 10% difference in sensitivity for small bowel disease extent between MRE and IUS; sample size calculation of 210 patients [20]. No formal power calculation was performed for this retrospective analysis. We imputed missing data where disease was not seen by either or both imaging modality, using a worst-case scenario assuming any missed disease was inactive for all categorical variables, with mural thickness assumed to be 1 mm and disease length 0 cm. We took this approach to account for potential bias when just considering disease identified by both modalities (Fig. S1).

Thereafter, we performed an analysis (complete case analysis) limited to patients where both MRE and IUS had identified terminal ileal disease, where activity parameters had been recorded for both modalities. Patients with missing data were excluded from this analysis.

### Additional subgroup analysis

Intermodality agreement of disease activity parameters, length of disease, and obstruction was also assessed separately in the following subgroups: (1) all patients with active terminal ileal disease according to the reference standard (regardless of whether identified by both imaging modalities) and (2) patients with inactive terminal ileal disease by the reference standard.

To better assess the potential clinical impact of any disagreement between modalities, we divided mean mural thickness into 3 categories (≤ 2 mm, > 2 to ≤ 4 mm, > 4 mm), and calculated limits of agreement, and how often measurements between modalities were within 2 mm of each other. Similarly, we divided mean disease length into ≤ 5 cm, > 5 to ≤ 10 cm, and > 10 cm, calculating limits of agreement and how many patients’ measurements between modalities were within 5 cm of each other.

### Statistical analysis methods

Frequencies and proportions were reported to summarise categorical variables. Medians and quartiles were reported for continuous variables. Bland–Altman plots with limits of agreement were used to assess agreement between MRE and IUS for disease length and mural thickness. Percentage agreement was used for categorical data. For mural thickness and disease length threshold subgroup group analysis, we reported the limits of agreement only to aid clinical interpretation of findings.

## Results

**Fig. 1:**
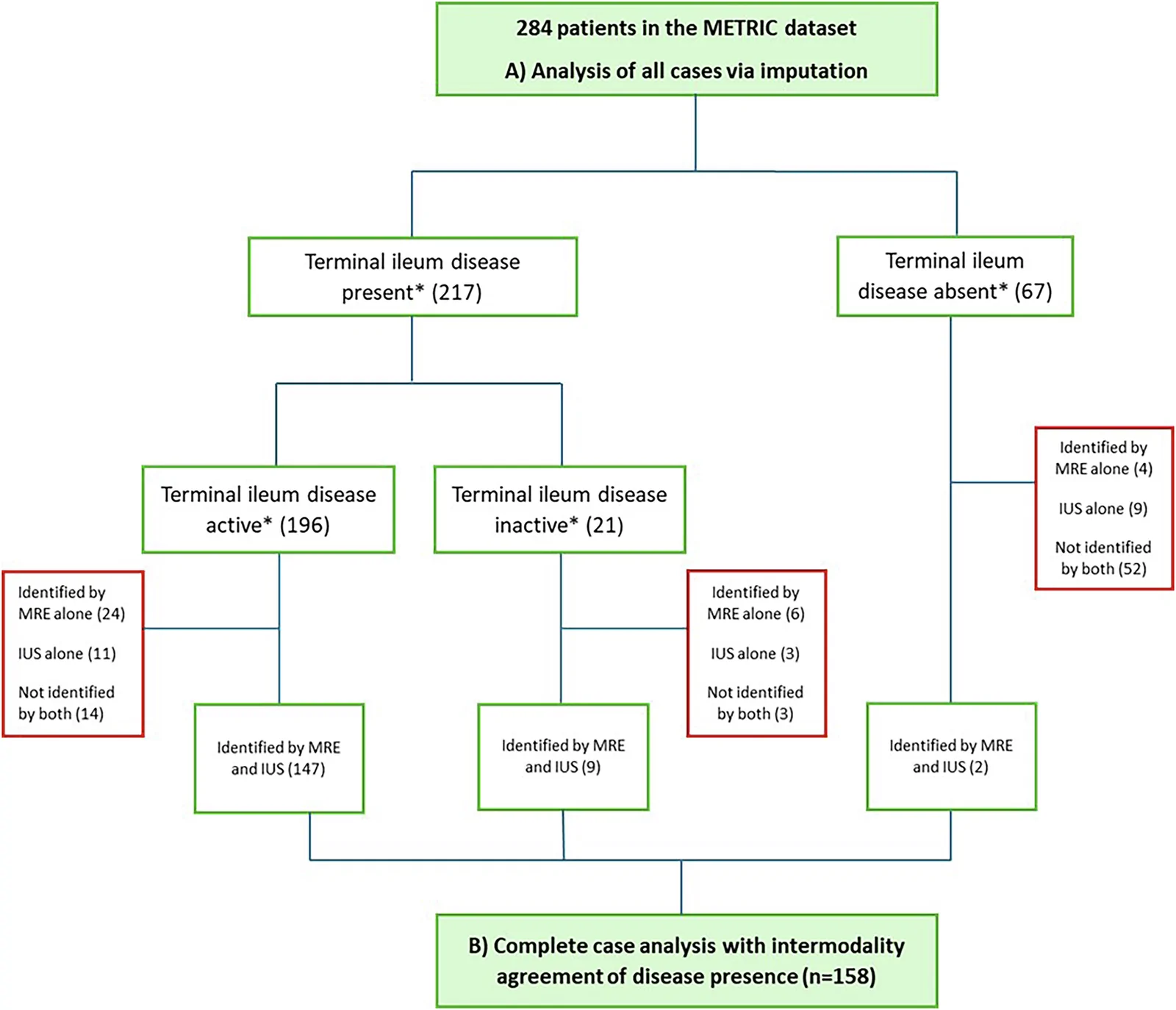
Flow of participants through the study and included in the analysis. * Deemed by the consensus panel reference standard. In 2/67 cases, terminal ileal disease was absent by reference standard, but MRE and IUS both identified disease; these cases were analysed as paired data

284 patients, median age of 36 years (IQR: 16–69), were included (133 (47%) new diagnosis; 151 (53%) suspected relapse). The flow of study participants is illustrated in Fig. 1, and baseline characteristics are shown in Table 2. Disease location was judged to be terminal ileal in 217 (76%) by reference standard. As previously reported, terminal ileal segmental visualisation for IUS was deemed poor in 7/133 (5.3%) cases in the new diagnosis cohort and 12/151 (7.9%) cases in the suspected relapse cohort. For MRE, this was 5/133 (3.8%) and 9 (6%), respectively [20].

**Table 2:** Baseline participant characteristics

Characteristic	Overall METRIC trial cohort, *n* = 284, *n* (%) or otherwise indicated	Paired MRE and IUS identified disease in the terminal ileum, *n* = 158, *n* (%) or otherwise indicated
Age (years)	36 (16–69)*	36 (23–56)*
Sex		
Male	130 (46)	77 (49)
Female	154 (54)	81 (51)
Patient type		
Newly diagnosed	133 (47)	84 (53)
Suspected relapse	151 (53)	74 (47)

* Median (Q1,Q3)

### Comparison of mural thickness measurement

**Fig. 2:**
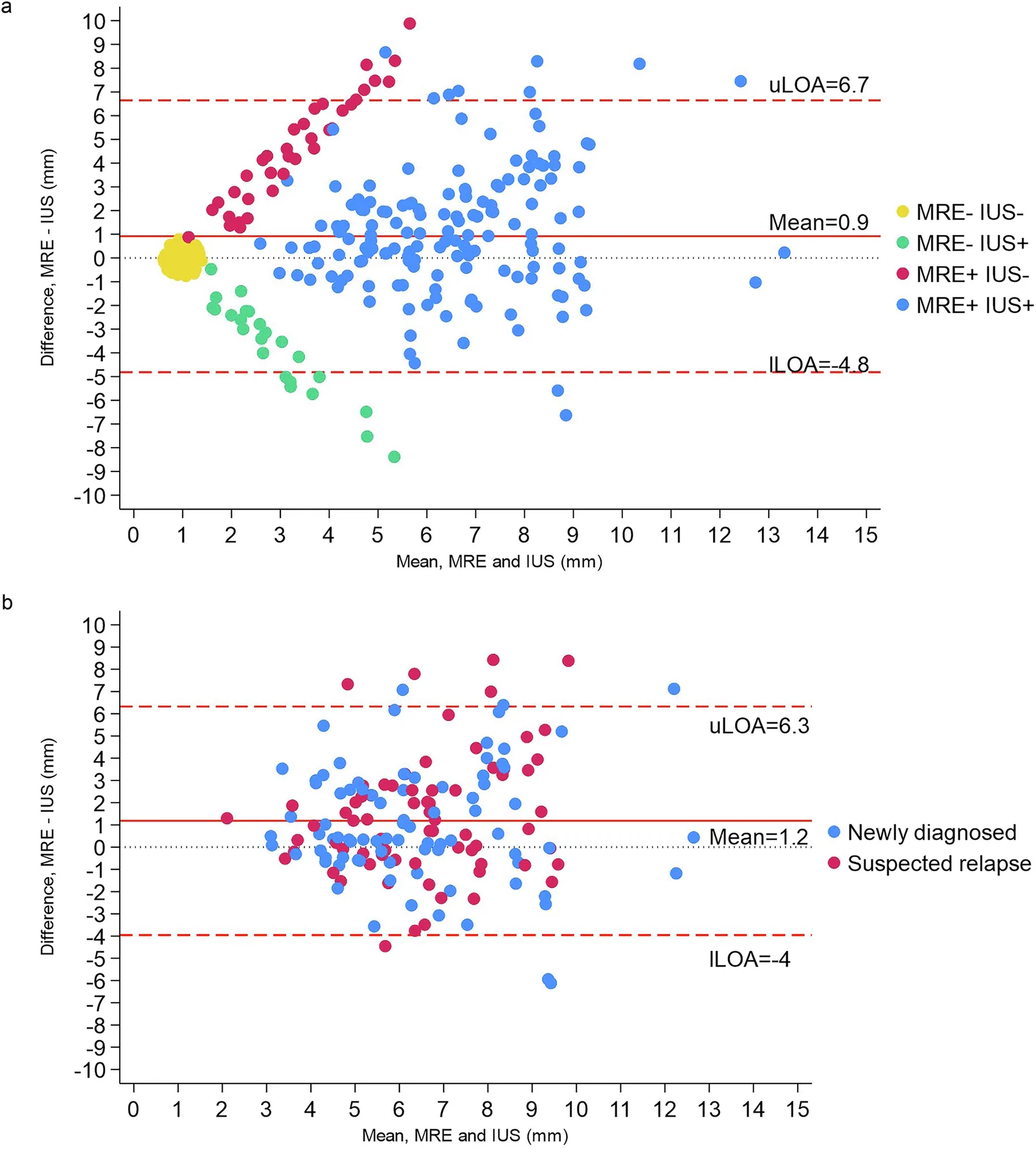
**a** Bland–Altman plot for agreement between MRE and IUS for mural thickness (millimetres) in the full cohort, *n* = 284. Imputation was performed for patients where either or both modalities did not identify disease, with the assumption being that disease was not present and inactive; a value of 1 was assigned. “-”: disease not detected on that modality, “+”: disease detected on that modality. **b** Bland–Altman plot for agreement between MRE and IUS for mural thickness (millimetres) where disease was detected on both modalities (complete case analysis), *n* = 155. Paired data were not available for 3 of the 158 patients included. uLOA, upper limit of agreement; lLOA, lower limit of agreement

Figure 2a shows agreement between MRE and IUS for terminal ileal mural thickness for all 284 patients. Figure 2b shows agreement for 155/158 patients with terminal ileal disease identified by both MRE and IUS (missing data in 3 patients).

Across all 284 patients, the mean mural thickness was 4.9 mm (95% CI: 4.5–5.3) on MRE and 4.0 mm (3.7–4.3) on IUS. Mean difference was 0.9 mm (95% LOA of −4.8 to 6.7 mm) between MRE and IUS measurements.

For the 155 patients with terminal ileal disease identified by both modalities, mean mural thickness was 7.1 mm (95% CI: 6.7–7.5) on MRE and 5.9 mm (5.6–6.3) on IUS. There was a mean difference of 1.2 mm (95% LOA of −4.0 to 6.3 mm) between MRE and IUS.

Table 3 illustrates agreement for the three threshold subgroups of mean mural thickness measurement. When the mean bowel mural thickness was > 2 and ≤ 4 mm, the 95% LOA ranged from −6.7 to 8.5 mm, and 24% (11/46) of measurements were within 2 mm of each other in the full cohort. For complete cases analysis, 95% LOA were −2.9 to 4.6 mm, and 85% (11/13) of measurements were within 2 mm. At > 4 mm mean mural thickness, 95% LOA were −4.9 to 7.7 mm, and 61% (94/153) measurements were within 2 mm for the full cohort (−4.0 to 6.5 mm and 66%, 94/142 for the complete cases analysis).

**Table 3:** Comparison of mean mural thickness and disease length measurements by subgroup

	Full cohort including imputed data (*n* = 284)		Complete case analysis (*n* = 155)	
Subgroup of mural thickness (mean measurement)	Measurements with ≤ 2 mm difference between modalities % (*n*/*N*)	95% LOA, mm	Measurements with ≤ 2 mm difference between modalities % (*n*/*N*)	95% LOA, mm
≤ 2 mm	100 (85/85)	−1.6 to 1.8	0	n/a
> 2 to ≤ 4 mm	24 (11/46)	−6.7 to 8.5	85 (11/13)	−2.9 to 4.6
> 4 mm	61 (94/153)	−4.9 to 7.7	66 (94/142)	−4.0 to 6.5

	Full cohort including imputed data (*n* = 284)		Complete case analysis (*n* = 156)	
Subgroup of disease length (mean measurement)	Measurements with ≤ 5 cm difference between modalities % (*n*/*N*)	95% LOA, cm	Measurements with ≤ 5 cm difference between modalities % (*n*/*N*)	95% LOA, cm
≤ 5 cm	92 (123/134)	−5.7 to 6.3	100 (20/20)	−3.2 to 2.4
> 5 to ≤ 10 cm	69 (41/59)	−15.1 to 14.9	82 (41/50)	−9.2 to 9.7
> 10 cm	41 (37/91)	−37.6 to 39.6	43 (37/86)	−36 to 38

95% limit of agreement (LOA) are presented alongside the percentage and frequency of cases in each threshold subgroup. Analysis was performed for both the complete case cohort and the full cohort with imputed data

### Comparison of disease length measurement

**Fig. 3:**
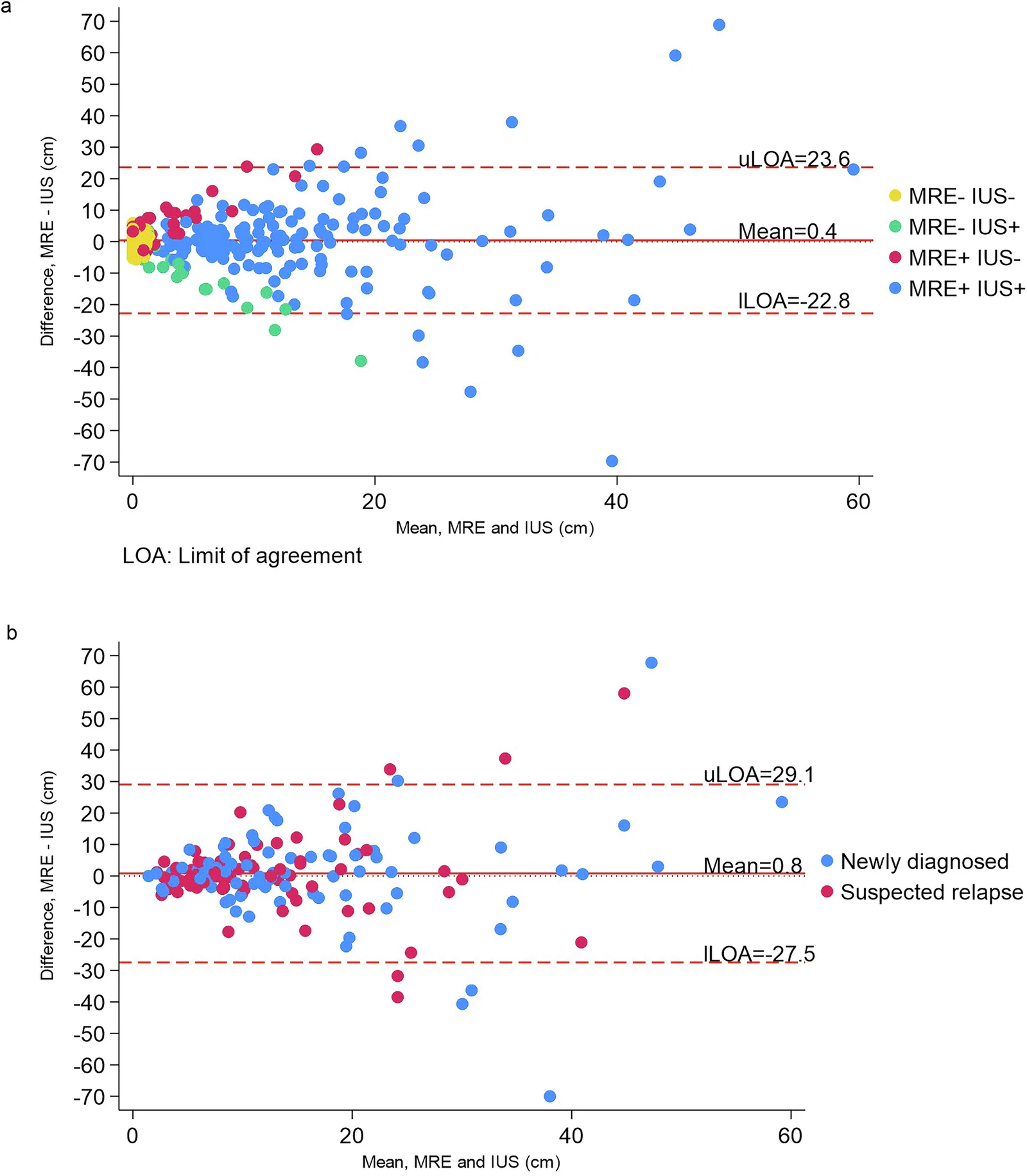
**a** Bland–Altman plot for agreement between MRE and IUS for disease length (centimetres) in the full cohort, *n* = 284. Imputation was performed for cases where either or both modalities did not identify disease, with the assumption being that disease was not present and inactive; a value of 0 was assigned. **b** Bland–Altman plot for agreement between MRE and IUS for disease length (centimetres), where disease was detected on both modalities (complete case analysis), *n* = 157. Paired data were not available for one patient of the 158 included. uLOA, upper limit of agreement; lLOA, lower limit of agreement

Across all patients, including imputation for missing data (*n* = 284), mean disease length was 9.1 cm (95% CI: 7.7–10.6) for MRE and 8.7 cm (95% CI: 7.4–10.0) for IUS. 95% LOA spanned −22.8 to 23.6 cm (Fig. 3a).

Figure 3b illustrates agreement between MRE and IUS for the measurement of longitudinal disease length in the terminal ileum, for the 157/158 patients where disease was detected on both modalities and available. Mean disease length in this subgroup for MRE was 15.1 cm (95% CI: 12.4–16.2) and 14.3 cm (95% CI: 13–17.2) for IUS. 95% LOA spanned from −27.5 to 29.1 cm.

For the disease length subgroups (Table 3), agreement was better at lower mean measurements; at ≤ 5 cm, the 95% LOA was −5.7 to 6.3 cm for the full cohort and −3.2 to 2.4 cm for complete case analysis. At ≤ 5 cm, measurements were within 5 cm in 92% (123/134) cases and 100% (20/20) respectively, and at > 5 and ≤ 10 cm, were 69% (41/59) and 82% (41/50) respectively. By contrast, for measurements > 10 cm, 41% and 43% were within 5 cm for the full cohort and complete cases.

### Agreement for parameters of disease activity and obstruction

**Fig. 4:**
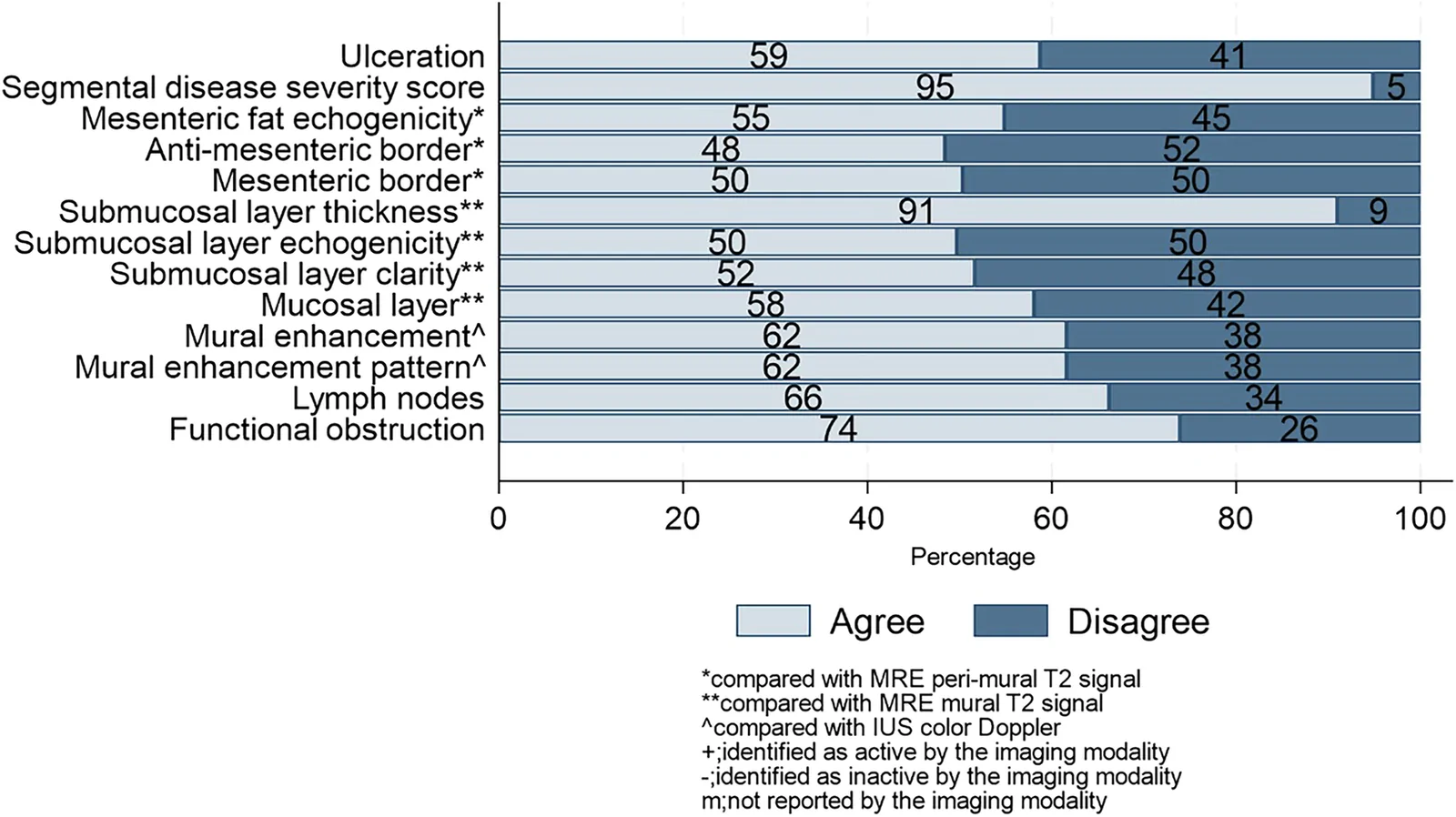
Agreement for MRE and IUS for assessment of disease activity parameters and functional obstruction. Where measurements were not possible in all 158 patients and/or incomplete data on either or both modalities, the number included for each parameter is indicated. Assessment of lymph nodes was possible in all 284 METRIC trial patients, as was assessed irrespective of whether luminal disease was identified

Figure 4 and Table 4 show agreement between the various parameters related to disease activity in patients with disease identified by both MRE and IUS (complete case analysis). Increased mural T2 signal (i.e., oedema on MRE) demonstrated the highest agreement with submucosal layer thickening on IUS (91%, 141/155). There was low intermodality agreement between other potentially equivalent IUS signs of increased mural T2 signal (50–58%), such as submucosal clarity and signs of perimural oedema (48–55%). There was modest agreement for mural enhancement level and pattern (62%) with IUS colour Doppler, ulceration (59%), and lymph node enlargement (66%).

**Table 4:** Percentage agreement between MRE and IUS assessment of disease activity parameters and functional obstruction

	*N* (% agreement, 95% CI)
Parameter	
Ulceration	91 (59, 51–67)
Segmental disease severity score	147 (95, 90–98)
Mesenteric fat echogenicity*	85 (55, 47–63)
Anti-mesenteric border*	75 (48, 40–57)
Mesenteric border	78 (50, 42–58)
Submucosal layer thickness**	141 (91, 85–95)
Submucosal layer echogenicity**	77 (50, 42–58)
Submucosal layer clarity**	80 (52, 43–60)
Mucosal layer**	90 (58, 50–66)
Mural enhancement^	93 (62, 53–69)
Mural enhancement pattern^	93 (62, 53–69)
Lymph nodes	188 (66, 60–72)
Functional obstruction	116 (74, 66–81)

*CI* confidence interval

* Compared with MRE perimural T2 signal

** Compared with MRE mural T2 signal

^ Compared with IUS colour Doppler

Conversely, the global segmental disease severity score (Table 1) demonstrated high intermodality agreement (95%, 147/155). There was also moderate agreement between MRE and IUS for the diagnosis of obstruction (74%, 116/157).

For patients with active terminal ileal disease by reference standard, 67% (131/196) had both increased mural T2 signal on MRE and submucosal thickening on IUS. Ulceration was reported by both modalities in only 21% (41/196, Fig. S2). Increased mural enhancement on MRE was seen in 69% (135/196) patients versus 44% (86/196) with increased colour Doppler signal on IUS.

For patients with inactive disease by reference standard (*n* = 21, Fig. S3), both mural T2 signal and submucosal thickening were absent in only one patient (5%).

## Discussion

In this post hoc review of patients recruited to the METRIC trial, we found that a global judgement of segmental disease severity exhibited good intermodality agreement in patients with disease identified on both modalities. In contrast, there was relatively poor intermodality agreement overall for mural thickness and disease length, both for the full cohort and for those with disease identified by both MR Enterography and Intestinal Ultrasound. Increased mural T2 signal, an important sign of disease activity, demonstrated the highest levels of agreement with submucosal thickening on IUS. There was poor agreement for other signs of mural and perimural oedema, and modest agreement for correlates of hypervascularity, ulceration, and lymph node enlargement.

MRE and IUS are both advocated in multidisciplinary guidelines as appropriate first-line investigations for CD diagnosis and follow-up, including evaluating therapeutic response [1, 2, 21]. The METRIC trial reported that MRE and IUS are both highly sensitive and specific for active small bowel disease in both newly diagnosed and suspected relapse patients [20]. Requirement for reliable intermodality comparisons is increasingly common in clinical practice, driven partly by the appropriate expansion of IUS services, particularly to monitor treatment response. Modality choice is not standardised, and many patients typically undergo an often arbitrary combination of MRE and IUS, which may have important implications for “treat-to-target” strategies if modalities are not interchangeable [4].

Many prior studies have not included all patients (i.e., including disease missed on either or both modalities). This introduces potential bias by restricting analysis to disease identified by both modalities, which may be intrinsically different from missed disease. To analyse the full cohort, we employed imputation, which assumed that any missed disease was inactive. Therefore, full cohort analysis presents a theoretical worst-case scenario for agreement.

Importantly, we found poor agreement for mean mural thickness measurements of > 2 and ≤ 4 mm in the full cohort (24% of modality measurements were with 2 mm), although 85% of measurements were within 2 mm when disease was seen on both modalities. There is potentially less clinical impact for lower agreement at greater mural thicknesses, e.g., 7 versus 9 mm, since disease is likely to be active in any event. However, small differences at lesser thicknesses may have a greater impact, given that 3 mm is the consensus threshold for normal mural thickness. Disagreement at this lower range of values could impact assessing early disease response, calculating disease activity scores, and even the incorrect classification of disease active versus inactive. This may lead to incorrect treatment decisions in a treat-to-target approach.

Agreement was greater for shorter disease lengths (≤ 5 cm) but poorer at longer lengths, particularly > 10 cm. We found high levels of agreement for segmental judgements of disease severity, suggesting the overall impression formed by the observer is similar using either modality. We found that increased mural T2 signal showed good agreement with IUS submucosal thickening only, and not submucosal clarity or echogenicity. Submucosal thickening alone is generally considered a sign of chronic, inactive disease. However, in an imaging surgical specimen correlation study, Bhatnagar et al also reported a statistically univariate significant association between submucosal thickening and histopathological disease activity [22]. Although measurements such as mural thickness and submucosal changes remain an important part of overall assessment, our data suggest that parameters from different imaging modalities should be compared cautiously in isolation. Our findings indicate that a holistic, qualitative approach incorporating validated parameters may be a more robust approach in routine clinical practice, particularly when there is expertise in using both modalities.

Previous studies have methodological limitations. Many present correlation coefficients alone without important agreement data which restricts inference of the clinical implications of measurement disagreement and comparison with our findings. Bland–Altman graphs allow interpretation of the size and direction of any disagreement across the whole range of measurements. In contrast to our findings, prior studies of small cohorts with limited readers have reported high levels of intermodality agreement for bowel mural thickness and, in most, disease length.

One of the largest prior studies, by Horje et al, reported a mean difference of 0.07 mm for wall thickness in 30 terminal ileal cases but without 95% LOA, impairing interpretation of agreement. Wilkens et al reported a 0.22 mm mean difference, but LOA of −4.3 to 3.9 for mural thickness in 25 moderate to severe CD patients [15]. Using a single MRE interpreter, Dillman et al reported “fair to moderate” correlation for disease length but “substantial” correlation for mural thickness in 29 newly diagnosed paediatric patients [14]. A prospective study of 120 small bowel CD patients found that IUS underestimated disease length by an average of 30% and “seemed to be significantly less accurate” [16], similar to our findings.

Unlike the current study, Horje et al found agreement for “stratified wall appearance” in 85% (89/105) [13]. Martinez et al reported a positive correlation between colour Doppler flow and mural enhancement, and subjective peri-enteric changes in 30 CD [18]. Modest agreement for mural enhancement and/or “comb sign” on MRE and any level of increased colour Doppler signal was also reported in another study of 43 ileal CD cases [17]. It is difficult to draw conclusions from the limited analysis presented in either study; we found poor to modest agreement for mural enhancement and pattern.

Our study has several strengths. METRIC used a large number of independent, blinded practitioners (*n* = 28) across 8 centres. Disease activity would be unlikely to change significantly between MRE and IUS as they were acquired within 4 weeks of each other, although some change cannot be entirely excluded. METRIC used a robust reference standard of a multidisciplinary consensus panel, important as intermodality agreement varies depending on activity level. We mapped 14 prespecified IUS activity variables to 10 specific MRE correlates. We also assessed the full cohort using imputation rather than risk bias by analysing only those patients where both modalities detected disease. We report 95% limits of agreement, which are more appropriate and clinically interpretable than correlation coefficients.

Our study also has limitations. METRIC predated established IUS scoring systems. Instead, we used a simple, global segmental disease severity score incorporating key signs of activity, many included in current scoring systems [23]. The retrospective nature of the study meant that we could not control for potential confounders, and findings are limited to terminal ileal disease location in new diagnosis and suspected relapse cohorts. Our findings suggest persistent abnormality on MRE and/or IUS even in the inactive disease subgroup (Fig. S3) but was an unpowered exploratory analysis. Our initial findings need prospective confirmation as well as external validation, and studies are currently underway. Previous imaging would normally be available for comparison when assessing treatment response, potentially allowing better registration between segments. The MANTRA study (clinicaltrials.gov: NCT06800326) is recruiting CD patients undergoing both IUS and MRE for treatment follow-up, and radiologists are interpreting varying combinations of baseline and follow-up imaging to assign the ECCO-ESGAR response group [1], in addition to specific imaging parameters and updated scores of activity.

In summary, we found reasonable agreement between MR Enterography and Intestinal Ultrasound of a global segmental disease severity score in Crohn’s disease patients with disease identified on both modalities. In contrast, mural thickness and disease length showed poor intermodality agreement in our cohort. Increased mural T2 signal showed good agreement with submucosal layer thickening on IUS. Our findings suggest caution when some commonly assessed signs of disease are compared between MRE and IUS in isolation. Studies to further validate our findings and on the clinical impact of intermodality comparisons in the treatment response setting are awaited, to further inform optimal, standardised imaging selection and training.

## Supplementary information

Supplementary information

## Data Availability

Data generated or analysed during the study are available from the corresponding author upon request.

## Abbreviations

CD: Crohn’s disease
CI: Confidence interval
IQR: Interquartile range
IUS: Intestinal Ultrasound
LOA: Limits of agreement
MRE: Magnetic Resonance Enterography
