# Supplementary material for: Evaluating MR enterography and intestinal ultrasound for ileal Crohn’s disease activity: a multicentre agreement study
Source: Eur Radiol Abdomen. 2026 Sep 11;1(1):2. doi: 10.1007/s44501-026-00002-8 (PMC13602877; doi:10.1007/s44501-026-00002-8)
Supplement: Supplementary file 1 [file 44501_2026_2_MOESM1_ESM.pdf]

Supplementary figures

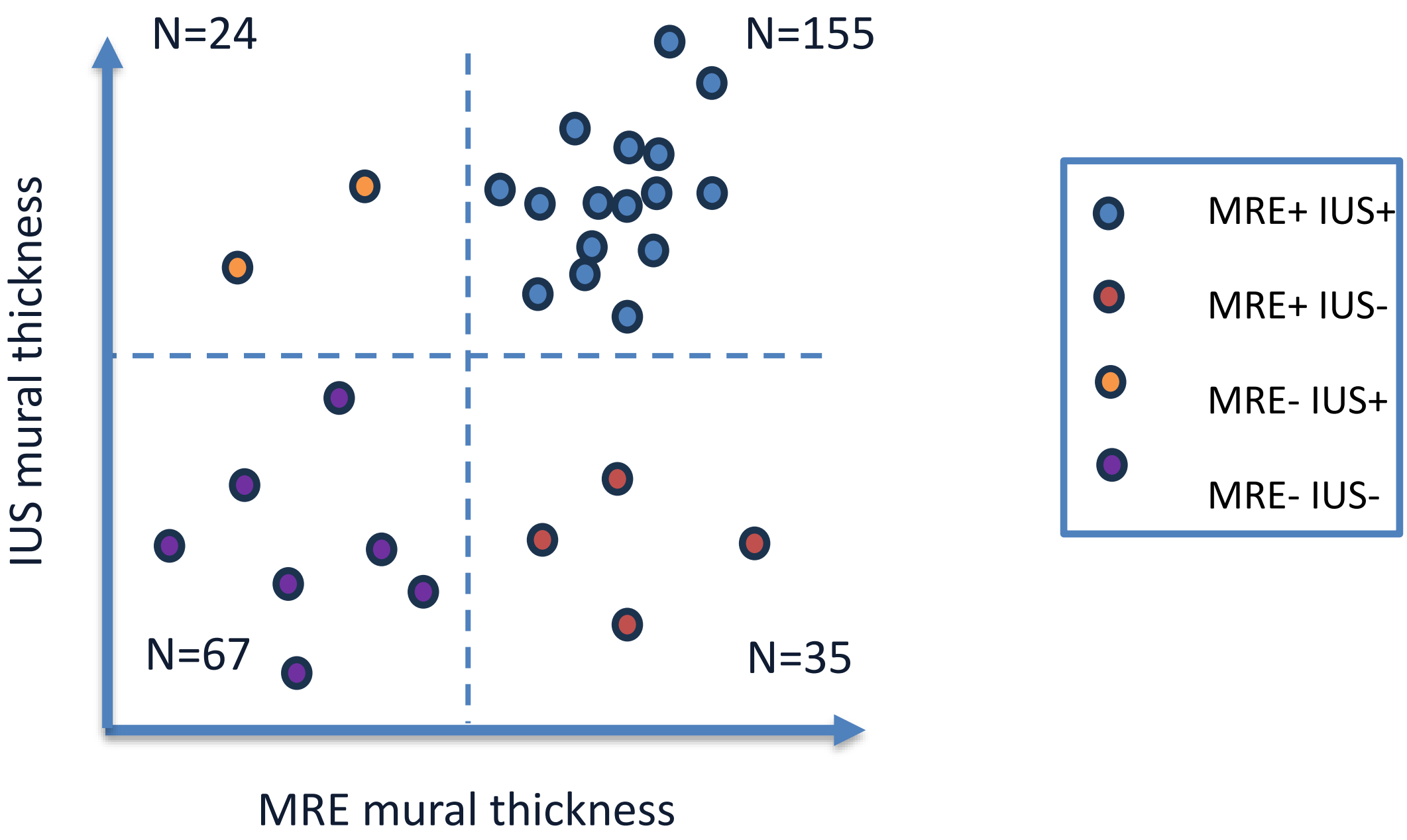

Figure S1: Complete case analysis via imputation. Illustration of the need for imputation to account for missing data where either or both modality did not identify disease and so detailed assessment of individual parameters was not performed. Analysis accounting for missing data avoids the potential bias of only analysing the subgroup with matched disease presence on MRE and IUS (complete case).

The above example demonstrates this point for mural thickness measurement. In 155 cases there was data available for when both MRE and IUS identified disease ("MRE+ IUS+"). However, this would ignore the 126/284 (44%) cases where either or both modality did not identify disease.

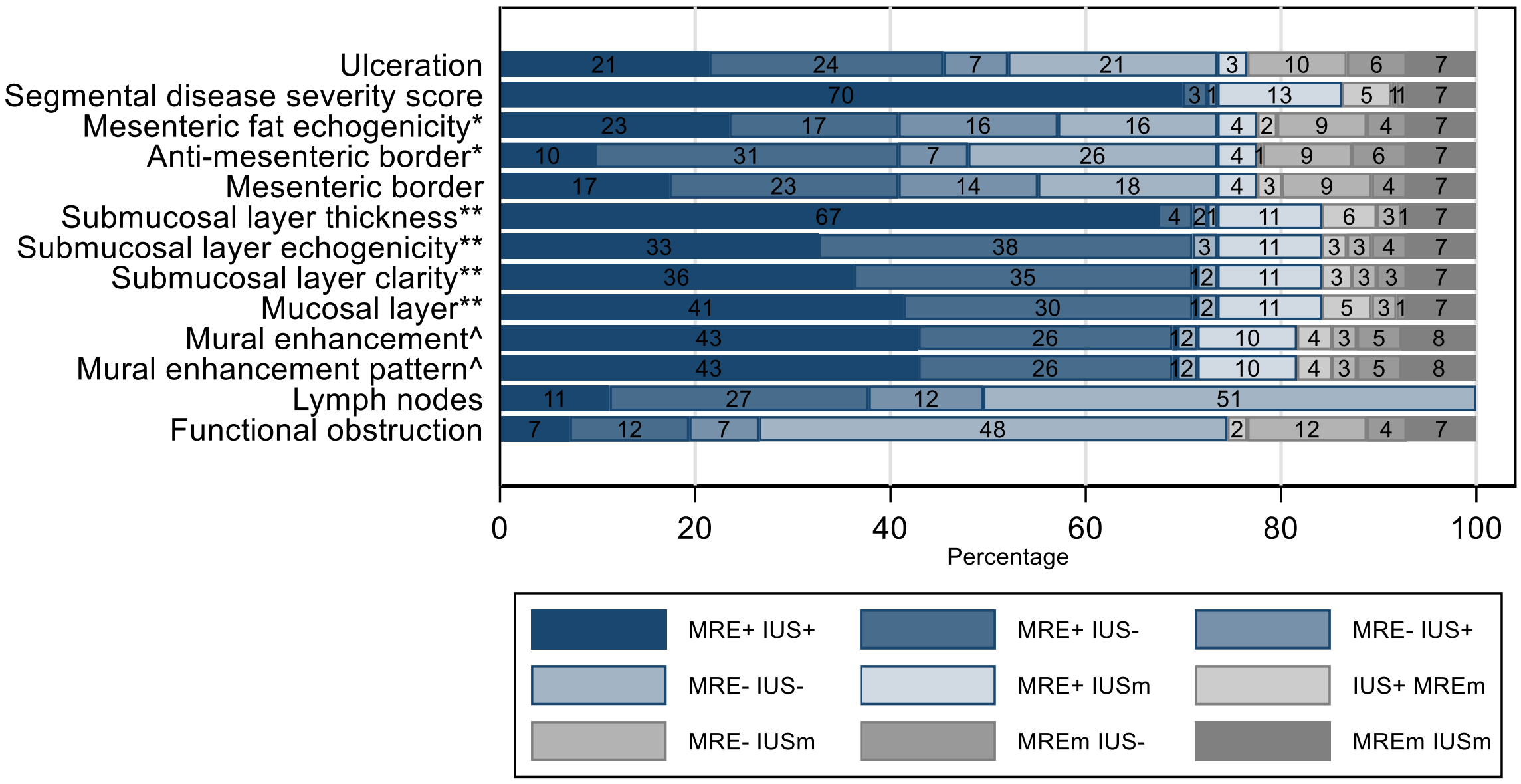

*compared with MRE peri-mural T2 signal
**compared with MRE mural T2 signal
^compared with IUS color Doppler
+;identified as active by the imaging modality
-;identified as inactive by the imaging modality
m;not reported by the imaging modality

Figure S2: Intermodality percentage agreement for disease activity parameters between MRE and IUS in patients with active disease in the terminal ileum defined by the multidisciplinary consensus panel reference standard (n=196). Agreement is reported separately for cases where either, both or neither modality reported active disease, and if the modality did not detect disease (m= missing data).

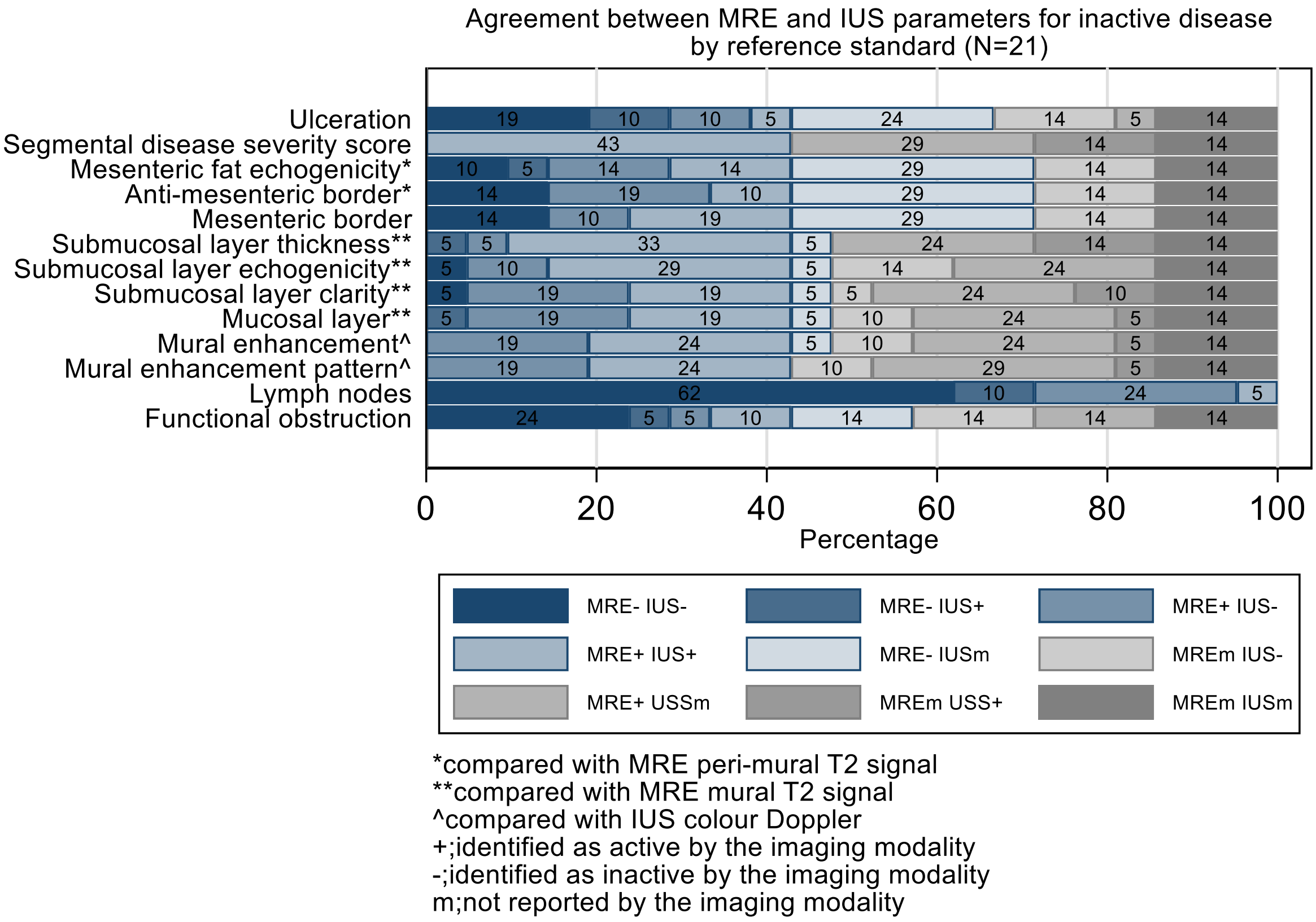

Figure S3: Intermodality percentage agreement for disease activity parameters between MRE and IUS in patients with inactive disease in the terminal ileum defined by the multidisciplinary consensus panel reference standard (n=21). Agreement is reported separately for cases where either, both or neither modality reported active disease, and if the modality did not detect disease (m= missing data).

Appendix 1: Case report form for ultrasound and imaging key

Radiologist Initials______________

USS platform______________________

Are you blinded to other clinical tests and investigations and patient clinical history (other than previous surgical history and new diagnosis or relapse cohort) ☐ **Yes** ☐ **No**

If N state what information/ test data you are aware of (eg barium FT)_____________________

Scan type (please circle)

Main Metric trial scan   Hydosonography substudy   Reader agreement substudy

If main Metric scan is this a repeat USS due to unblinded first USS examination? ☐ **Yes** ☐ **No**

**Hydrosonography sub study**			
**Performed using oral contrast given for same day MRE (circle)**	☐ **Yes**		☐ **No**
**Type of oral contrast**		**Volume ingested**	
**Time for ingestion**			

Case Report Form: Ultrasound Scanning: Scan quality

**Segment**	**Quality of segmental visualization to make correct diagnosis**<br>1-good/ 2-moderate/ 3-poor/ 4-N/A or excised				**If poor visualization, tick why**<br>1- overlying bowel gas/ *2- increased patient BM/ 3-Difficult anatomy***/ 4- patient pain/ 5-other (state)*				
	1	2	3	4	1	2	3	4	5
**duodenum**									
**Jejunum***									
**ileum**									
**Terminal ileum****									
**Caecum**									
**Ascending**									
**Transverse**									
**Descending**									
**Sigmoid**									
**Rectum**									

* small bowel from DJ flexure mainly to the left of a diagonal running from the RUQ to LLQ showing typical feathery fold pattern,

** last 10cm of ileum upstream of IV valve/anastomosis

***Eg low lying caecum

Ileocaecal valve identified ☐ **Yes** ☐ **No**

Case Report Form: Ultrasound Scanning Disease Assessment

**Overall disease assessment (to be completed for all patients)**						
	**Normal**		**Equivocal**		**Abnormal**	
**Confidence**	1 (disease definitely not present)	2 (disease probably not present)	3 (disease possibly not present)	4 (disease possibly present)	5 (disease probably present)	6 (disease definitely present)
**Any small bowel disease PRESENT?-** **tick confidence box**						
	1 (disease definitely not active)	2 (disease probably not active)	3 (disease possibly not active)	4 (disease possibly active)	5 (disease probably active)	6 (disease definitely active)
**If present (confidence score ≥3) is it ACTIVE? tick confidence box**						
	1 (disease definitely not present)	2 (disease probably not present)	3 (disease possibly not present)	4 (disease possibly present)	5 (disease probably present)	6 (disease definitely present)
**Any colonic disease PRESENT?** **tick confidence box**						
	1 (disease definitely not active)	2 (disease probably not active)	3 (disease possibly not active)	4 (disease possibly active)	5 (disease probably active)	6 (disease definitely active)
**If present (confidence score ≥3) is it ACTIVE? tick confidence box**						

Case Report Form: Ultrasound Scanning Other findings

**Lymphadenopathy (0-3)**	☐ **0**	☐ **1**	☐ **2**	☐ **3**
**Abnormal free fluid (Y/N)**	☐ **Yes**		☐ **No**	
**Abscess present**	☐ **Yes**		☐ **No**	
If yes please state size & location				
**Fistula present (circle all that apply)**	☐ **Yes**		☐ **No**	
If yes please circle location		Ileo-ilealIleo-colic entero-cutaneous ileo-vesical<br>colon-vesicaljejuno- jejunaljejuno-colic<br>Other (state________________________________)		
**Other small bowel diagnosis (eg adhesions, meckels, radiation enteritis etc**	☐ **Yes**		☐ **No**	
**If yes please state**				
**Extra enteric findings (eg aortic aneurysm gallstones, solid organ abnormality, phelgmon)**	☐ **Yes**		☐ **No**	
If yes please state				
**Are you recommending any further tests?**	☐ **Yes**		☐ **No**	
If yes please state which				

Case Report Form: Ultrasound Scanning Disease Presence						
Please complete for each segment			Confidence of disease PRESENCE			
	Normal		Equivocal		Abnormal	
**Segment**	1 (disease definitely not present)	2 (disease probably not present)	3 (disease possibly not present)	4 (disease possibly present)	5 (disease probably present)	6 (disease definitel y present)
**Duodenum (D)**						
**Jejunum (J)**						
**Ileum (I)**						
**Terminal ileum (TI)***						
**Caecum (C)**						
**Ascending colon (A)**						
**Transverse colon (T)**						
**Descending colon (D)**						
**Sigmoid (S)**						
**Rectum (R)**						

*throughout, if TI disease is contiguous for over 10cm count just as TI not TI and ileum

Case Report Form: Ultrasound Scanning Disease Activity

Confidence of disease ACTIVITY Please complete for each segmentif confidence scores 3-6 for disease presence above ie **present or equivocal**

		**Normal**		**Equivocal**		**Active**	
**Segment**	No disease (ie confidence scores 1 or 2 for disease presence)	1 (disease definitely not active)	2 (disease probably not active)	3 (disease possibly not active)	4 (disease possibly active)	5 (disease probably active)	6 (disease definitely active)
**Duodenum (D)**							
**Jejunum (J)**							
**Ileum (I)**							
**Terminal ileum (TI)**							
**Caecum (C)**							
**Ascending colon (A)**							
**Transverse colon (T)**							
**Descending colon (D)**							
**Sigmoid (S)**							
**Rectum (R)**							

Case Report Form: Ultrasound Scanning Disease Description Disease Site 1

PLEASE COMPLETE FOR EACH DISEASE SITE (DEFINED AS >3CM OF NORMAL BOWEL BETWEEN DISEASE SITES). USE ONE TABLE FOR EACH DISEASE SITE. ONLY RECORD SEGMENTS WHICH IF YOU HAVE A CONFIDENCE SCORE OF 3 OR MORE FOR DISEASE PRESENCE. USE SCORE DEFINITIONS AT THE START OF THIS CRF

Complete for equivocal or abnormal sites (ie confidence scores 3-6). Complete additional tables as required if multiple disease sites per segment

Location	Tick one location	Single Wall thickness (mm) thickest portion	Wall thickening (0-3)	Length of abnormal bowel (cm)	Stensois causing functional obstruction (0/1)	Mesenteric fat echogenicity	Anti-mesenteric border (0-4)	Mesenteric border (0-2)	Submucosal layer	Submucosal layer echogenicity (0-3)	Submucosal layer clarity (0/1)	Mucosal layer (0-2)	Ulceration (0-2)	Doppler vascular pattern axial section (0-2)	Peristaltic distension (o/1)	Does the segment contain established fibrosis (Y/N)	Segmental disease severity assessment (0-2)	Segment shows active disease (Y/N)
Duo																		
J																		
I																		
TI																		
C																		
A																		
Des																		
S																		
R																		

Full definitions of bowel segments

duodenum(D), jejunum(J), ileum(IL), terminal ileum(TI) and the colon into rectum(R), sigmoid(S), descending colon(DC), transverse(TC), ascending(AC) and caecum(C).

The J was defined as the proximal bowel lying largely to the left of a diagonal drawn from the right upper quadrant to the left lower quadrant demonstrating a typically feathery fold pattern. The TI (or neo-TI in the case of past resections) was defined as the last 10cm of small bowel upstream of the ileo-colonic junction. Contiguous disease involving the TI but extending beyond 10cm was classified as TI disease (as opposed to both TI and IL). Distinct sections of disease within a particular segment were defined as non-contiguous if 3cm or more of normal appearing small bowel was present between disease sites. Disease sections separated by less than 3cm of normal bowel were considered a single disease section (contiguous) for the purposes of data recording.

Ultrasound Imaging Key

Scoring guide to be used in conjunction with ultrasound CRF pictorial definitions.

**Bowel wall thickness score**	
**0**	0-3mm - Normal
**1**	3-6mm
**2**	6-9mm
**3**	>9mm

**Upstream dilatation score**	
**0**	None
**1**	Present

**Mesenteric fat echogenicity score**	
**0**	Normal
**1**	Focal hyperechoic without fat wrap (ie focal defined area of mesenteric fat of increased echogenicity without overall increase in volume of peri mural fat)
**2**	Focal hyperechoic with fat wrap (ie focal defined area of mesenteric fat of increased echogenicity with overall increase in volume of peri mural fat)
**3**	Stratified heterogeneous with fat expansion (chronic disease) - overall increase in volume of peri mural fat with maintained normal mesenteric stratification and no focal hyperechoic area)
**4**	Uniform hypoechoic (ie mesenteric fat of decreased echogenicity

Anti-mesenteric border score	
**0**	Well defined (smooth interface between bowel wall and mesenteric fat)
**1**	Ill defined (irregular Interface between bowel wall and mesenteric fat)

Mesenteric border score	
**0**	Well defined (smooth interface between bowel wall and mesenteric fat)
**1**	Generally ill defined (generally irregular Interface between bowel wall and mesenteric fat)
**2**	Focally ill defined (focal irregular Interface between bowel wall and mesenteric fat)

Submucosal layer score	
**0**	Normal
**1**	Thickened (increased thickness compared to submucosa of normal bowel in the same patient)

Submucosal layer echogenicity score	
**0**	Normal
**1**	Reduced (echogenicity lower than normal bowel in same patient)
**2**	Uniform increased (echogenicity greater than normal bowel in same patient)
**3**	Increased with bands (echogenicity greater than normal bowel in same patient with spoke wheel hypoechoic bands)

**Submucosal layer clarity score**	
**0**	Normal - well defined (smooth interface between submucosa and other wall layers)
**1**	Ill defined (irregular interface between submucosa and other wall layers)

**Mucosal layer score**	
**0**	Well defined (smooth interface between bowel wall and mesenteric fat)
**1**	Isolated thickened (increased thickness compared to mucosa of normal bowel in the same patient)
**2**	Thickened also in presence of submucosal thickening

**Ulceration score**	
**0**	None
**1**	Superficial-< 50% wall thickness
**2**	Deep-≥50% wall thickness

**Doppler vascular pattern imaged in axial section score**	
**0**	Well defined (smooth interface between bowel wall and mesenteric fat)
**1**	Increased focal (increased Doppler signal isolated to less than half the circumference on a trans-axial image) compared to normal bowel in the same patient)
**2**	Increased generalised (increased Doppler signal affecting more half the circumference on a trans-axial image compared to normal bowel in the same patient)

**Peristalsis related to stricturing score**	
**0**	Distension of the segment during peristaltic wave
**1**	Non distension of segment during proximal peristaltic wave

**Disease activity**
**At least one of: wall thickening/ focal hyperechoic mesentery (with or without fat wrap)/ isolated mucosal thickening /ill defined submucosal layer/ ill defined anti-mesenteric border/ Increased Doppler vascular pattern OR ulceration OR abscess**

**Segmental disease severity assessment score**	
**0**	None
**1**	Early-superficial ulceration and/or mild wall thickening/ mild increased vascularity
**2**	Advanced-transmural disease and/or fistulation and/or stricturing and/or cobblestoning/ and or wall oedema or significant thickening

**Lymphadenopathy (all measurements in short axis) score**	
**0**	None
**1**	Cluster all less than 1cm
**2**	1 or 2 nodes > 1cm
**3**	3 or more nodes greater than 1cm

Case Report Form-USS SCORING

Mesenteric Fat Echogenicity

0 - Normal

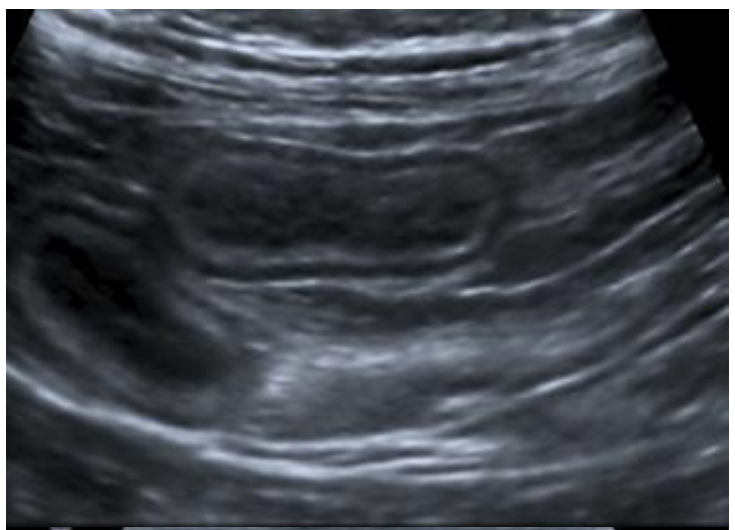

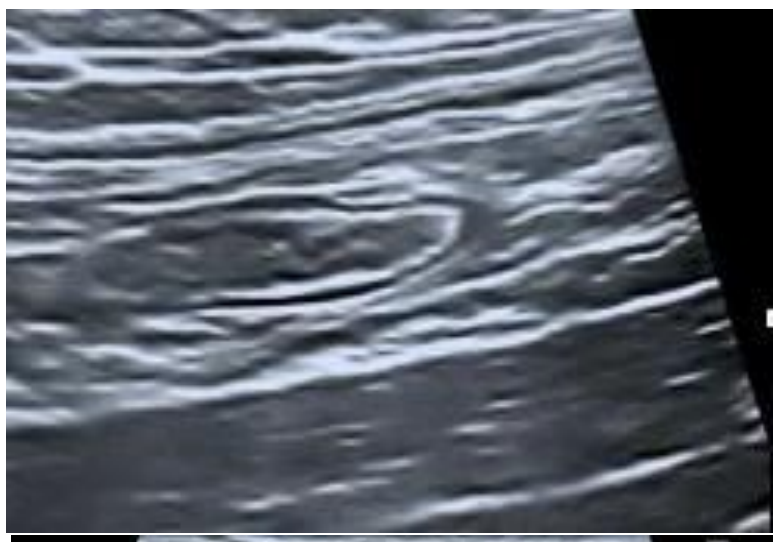

1 - Focal hyperechoic without fat wrap (ie focal defined area of mesenteric fat of increased echogenicity without overall increase in volume of peri mural fat)

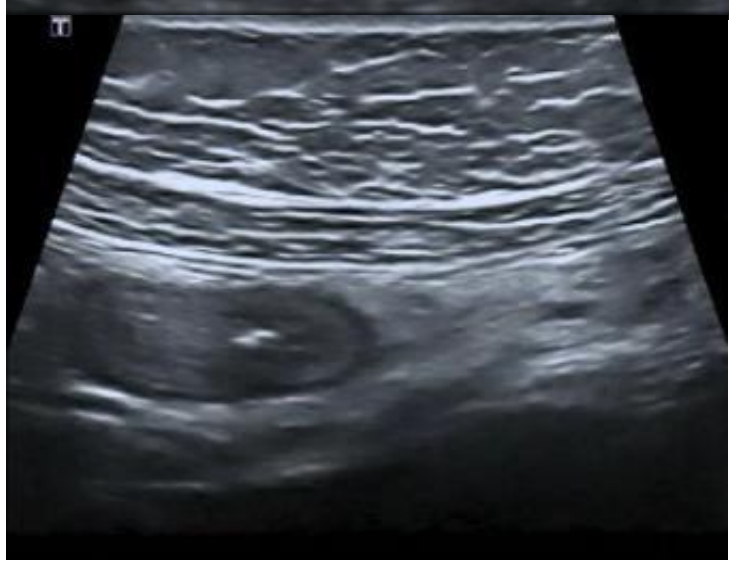

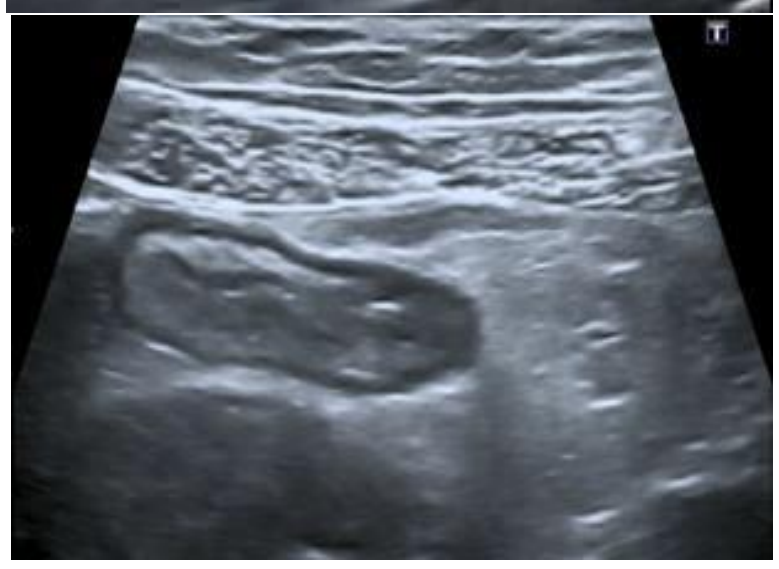

2 - Focal hyperechoic with fat wrap (ie focal defined area of mesenteric fat of increased echogenicity with overall increase in volume of peri mural fat)

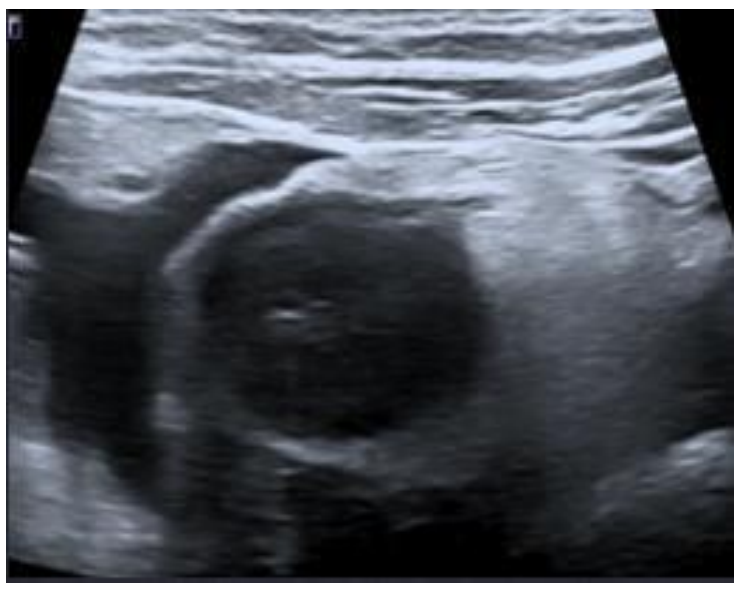

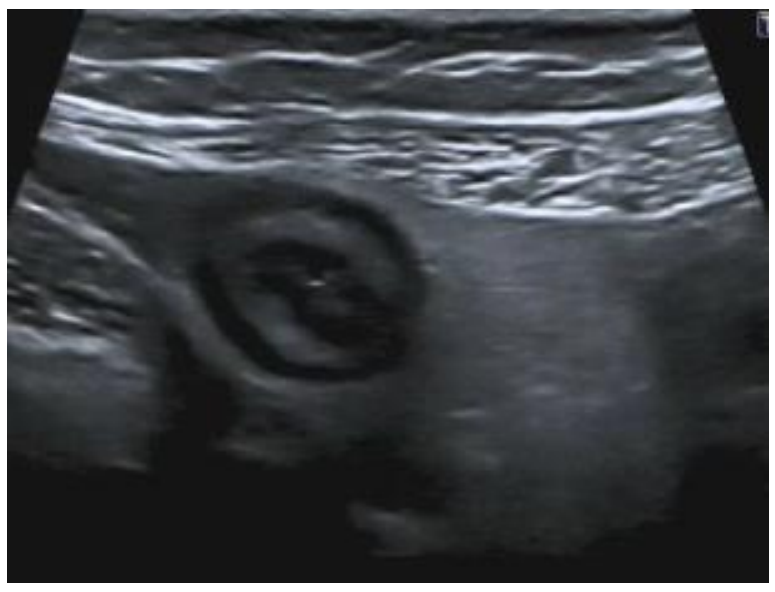

3 - Stratified heterogeneous with fat expansion (chronic disease) - overall increase in volume of peri mural fat with maintained normal mesenteric stratification and no focal hyperechoic area**)**

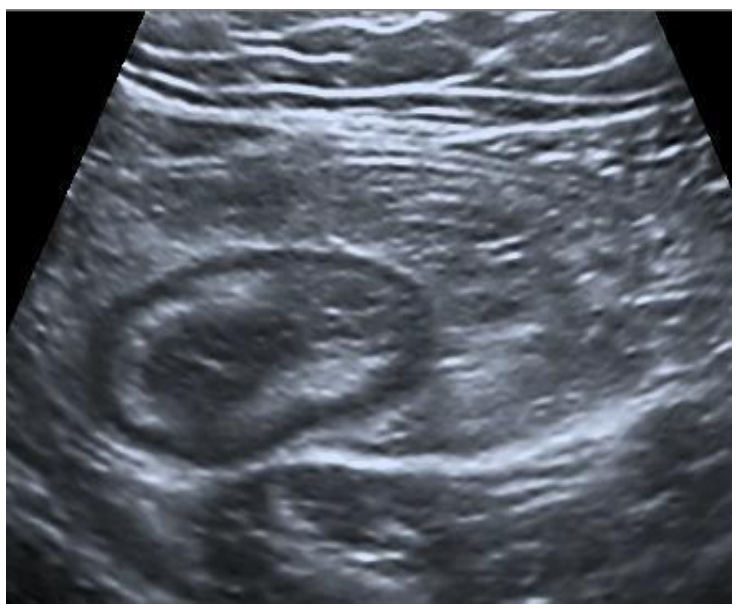

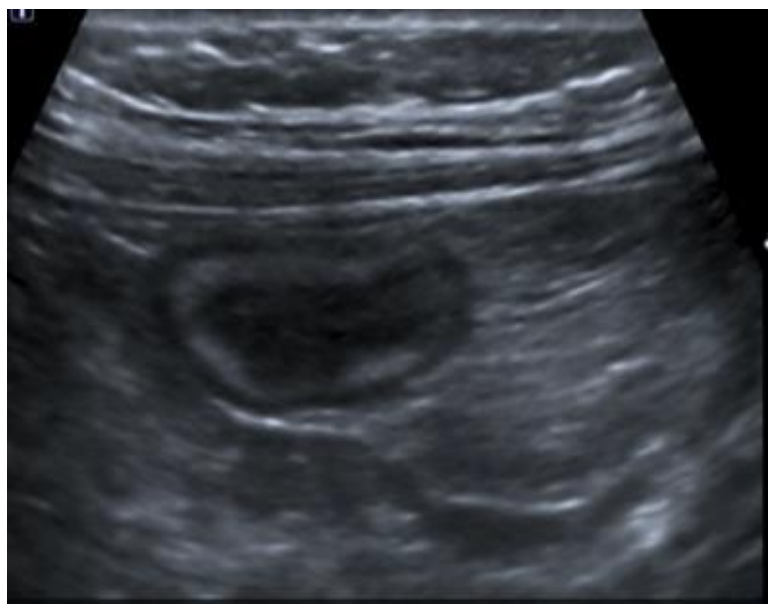

4 - Uniform hypoechoic (ie mesenteric fat of decreased echogenicity

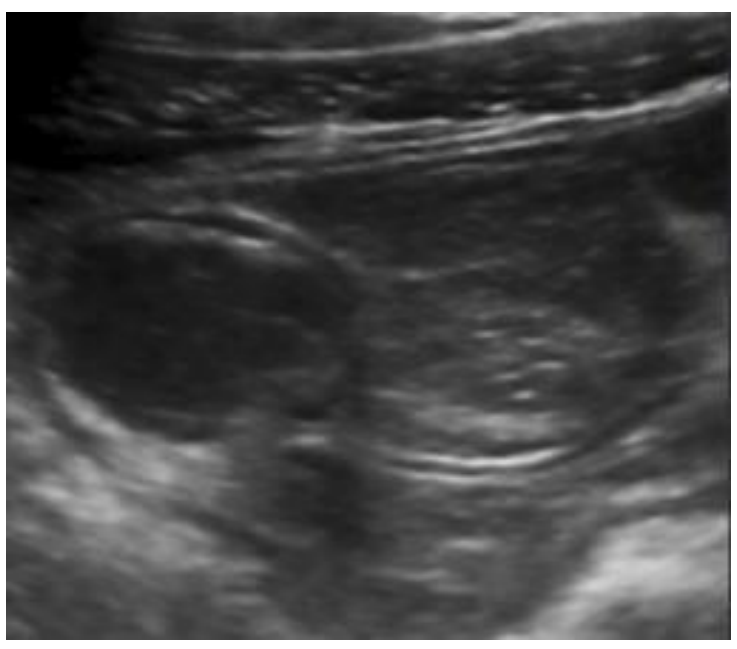

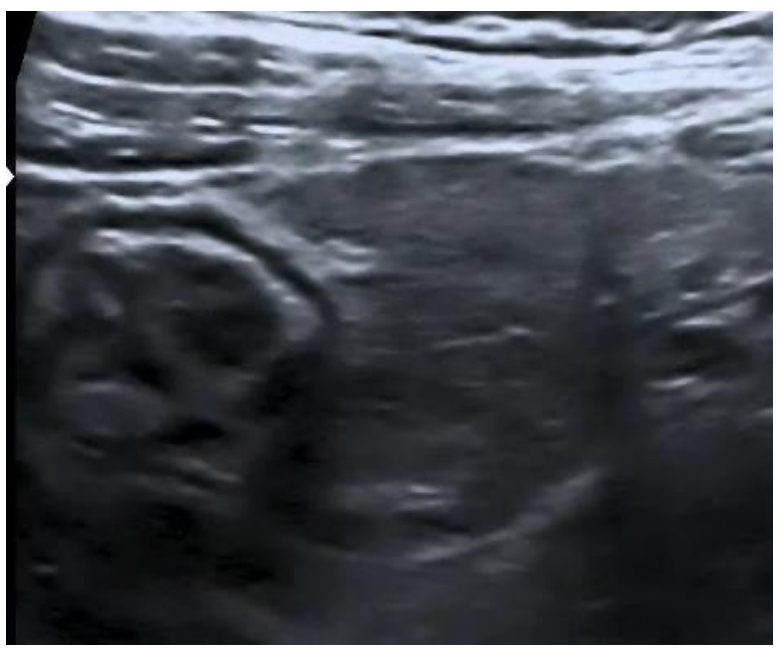

Case Report Form-USS SCORING

Anti-Mesenteric Border

Example 1

0 - Well defined (smooth interface between bowel wall and mesenteric fat)

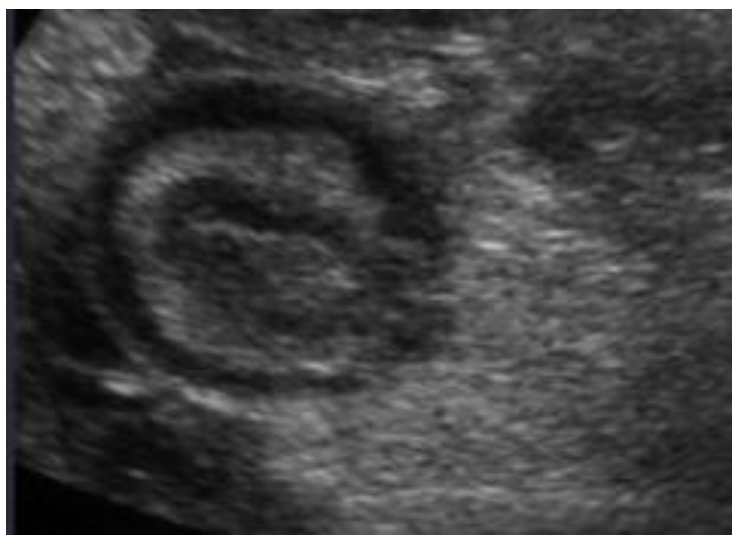

Post treatment

1 - Ill defined (irregular Interface between bowel wall and mesenteric fat)

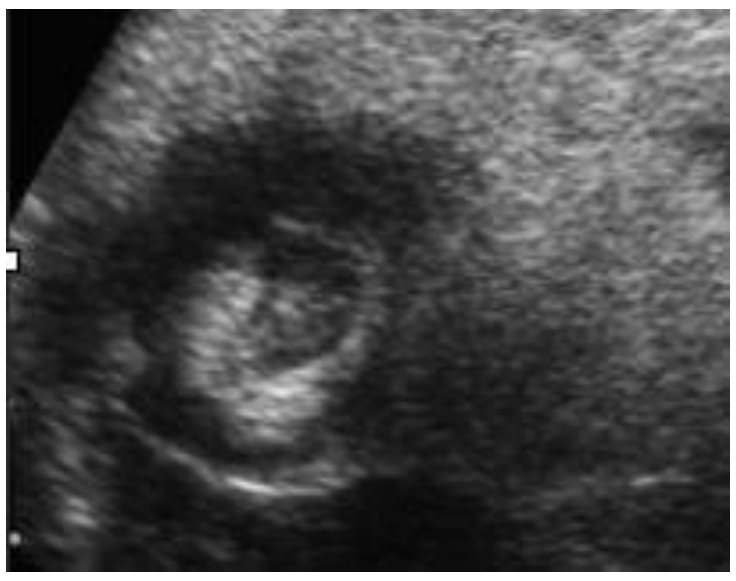

Pre treatment

Example 2

0 - Well defined (smooth interface between bowel wall and mesenteric fat)

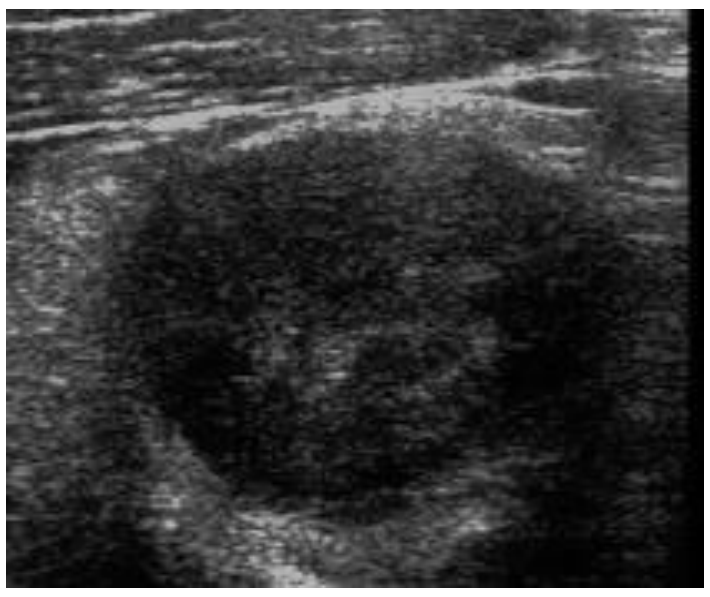

Post treatment

1 – Ill defined (irregular Interface between bowel wall and mesenteric fat

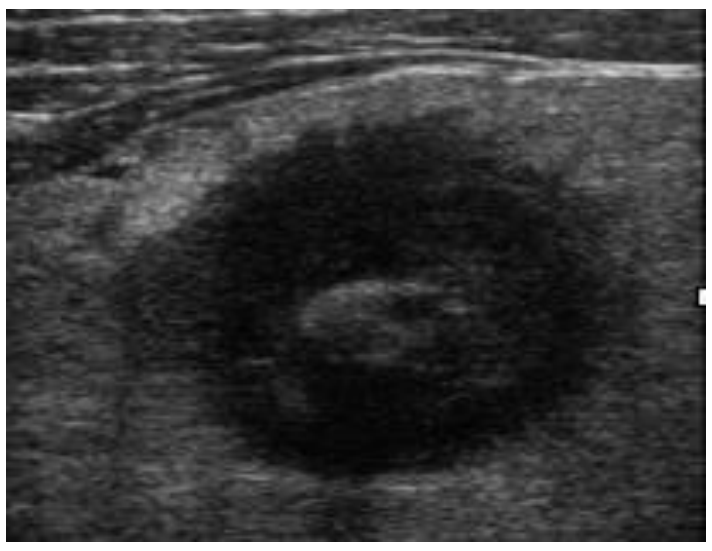

Pre treatment

Case Report Form-USS SCORING

Mesenteric Border

0 - Well defined

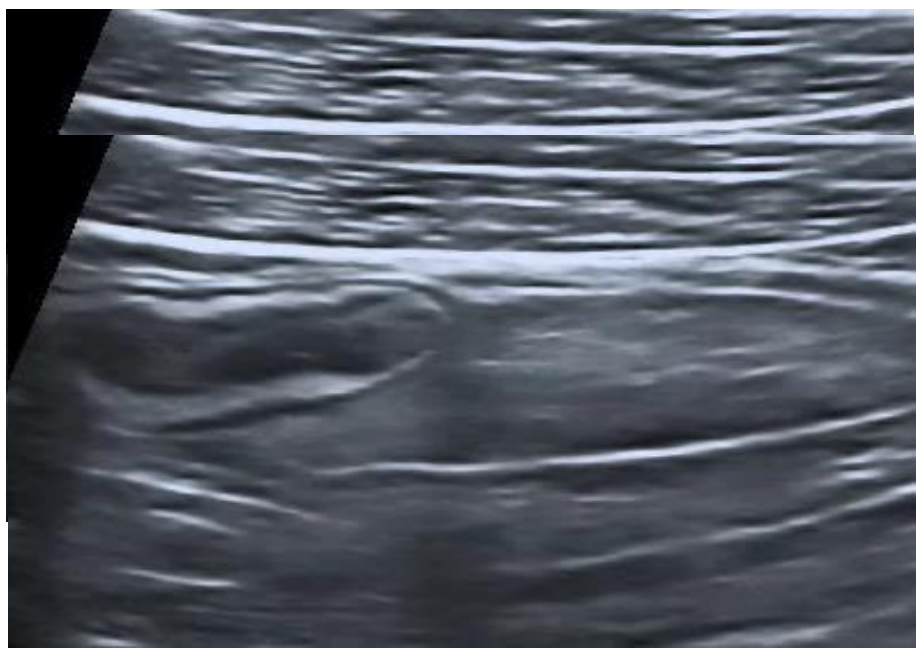

1 - Ill defined

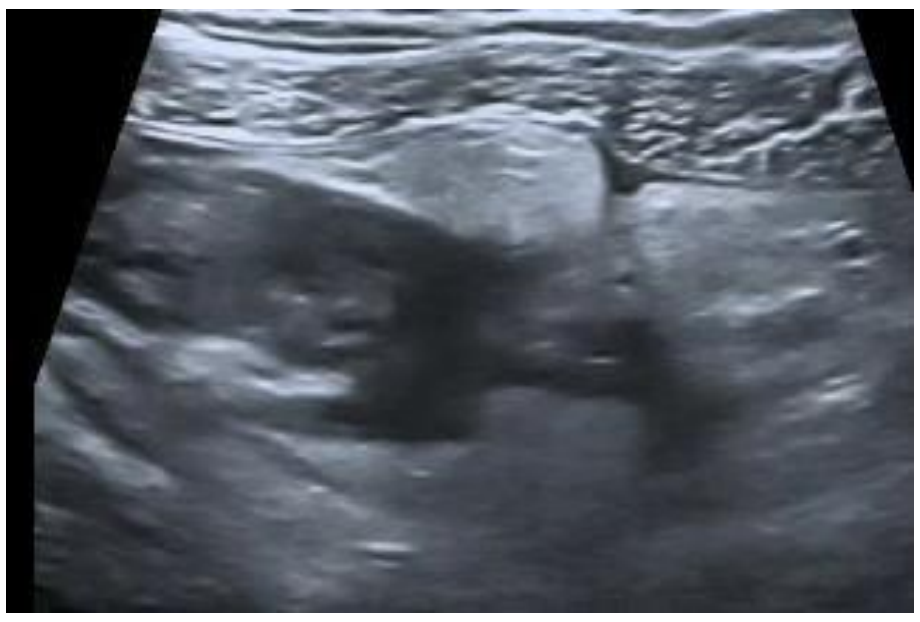

2 - Focal involvement

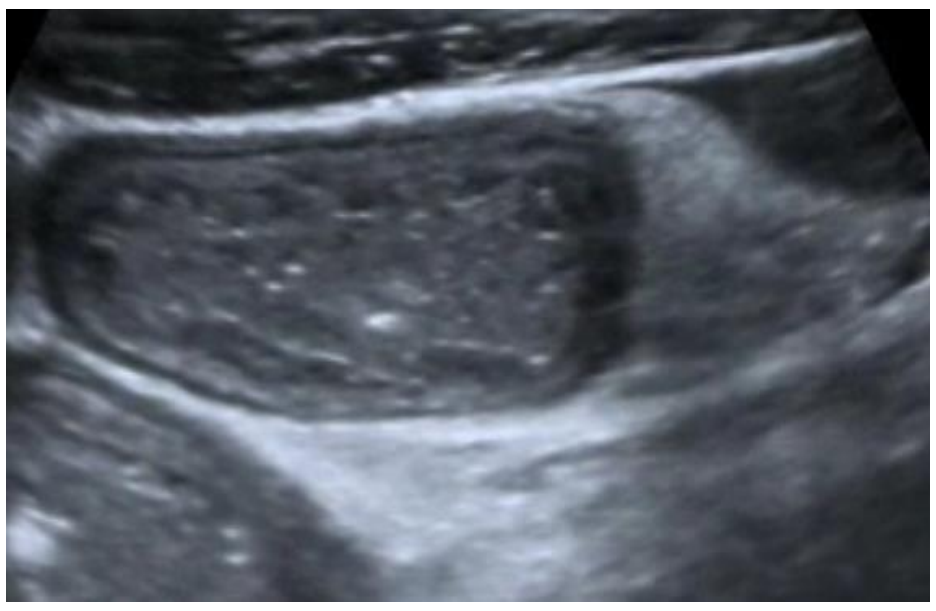

Submucosal layer

Ileostomy

0 - Normal

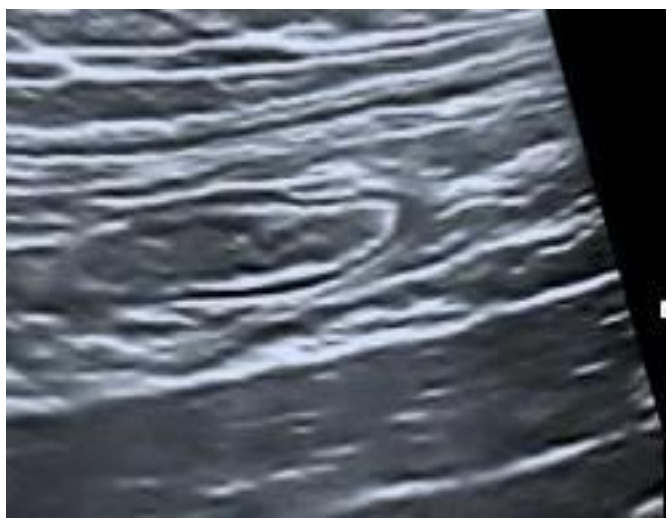

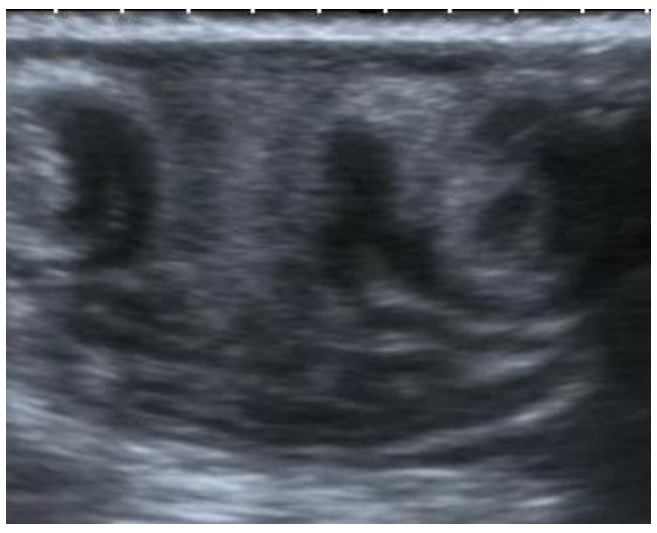

1 - Thickened (increased thickness compared to submucosa of normal bowel in the same patient

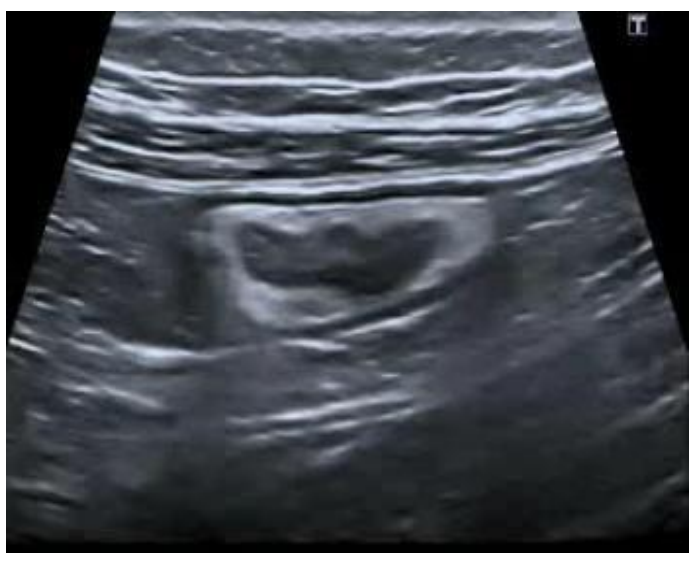

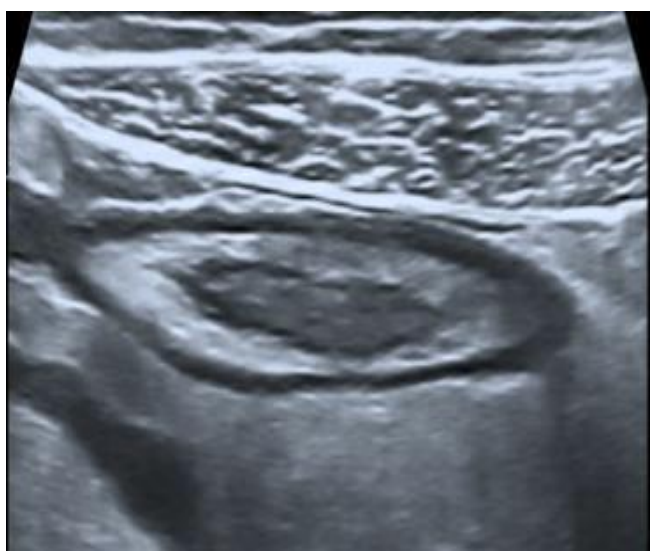

Case Report Form-USS SCORING

Sub-mucosal layer Echogenicity

0 - Normal

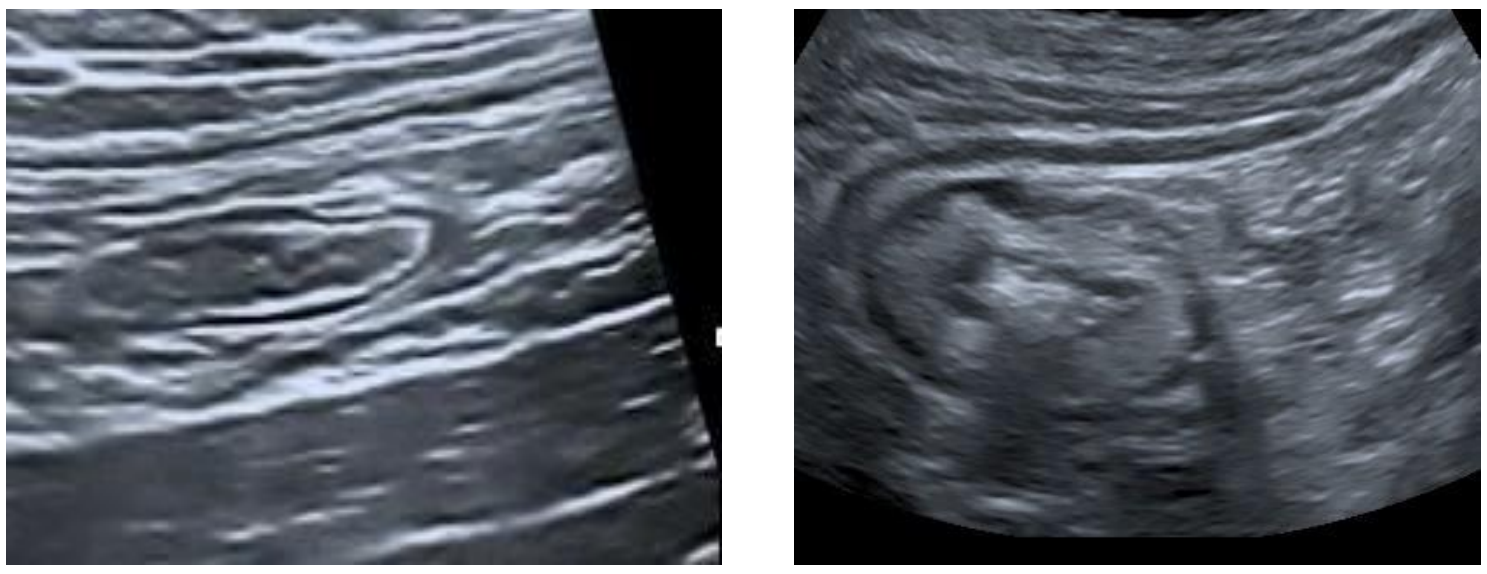

1 - Reduced (echogenicity lower than normal bowel in same patient)

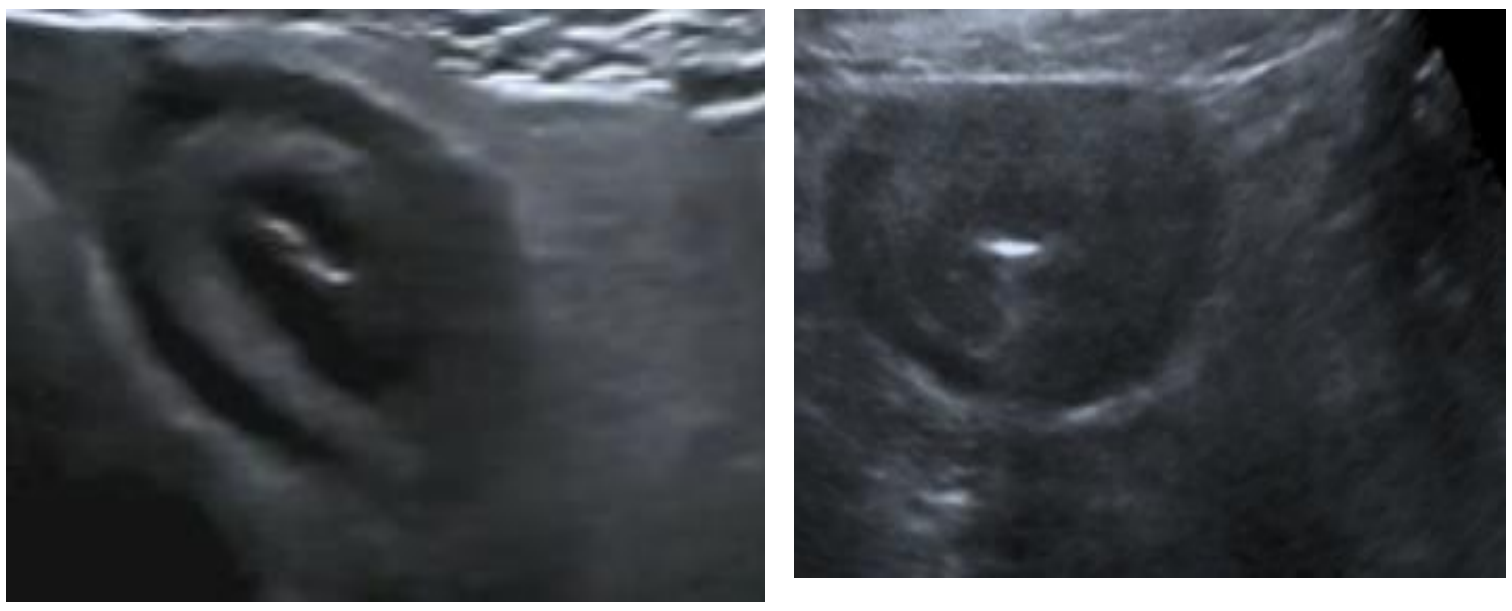

2 - Uniform increased (echogenicity greater than normal bowel in same patient)

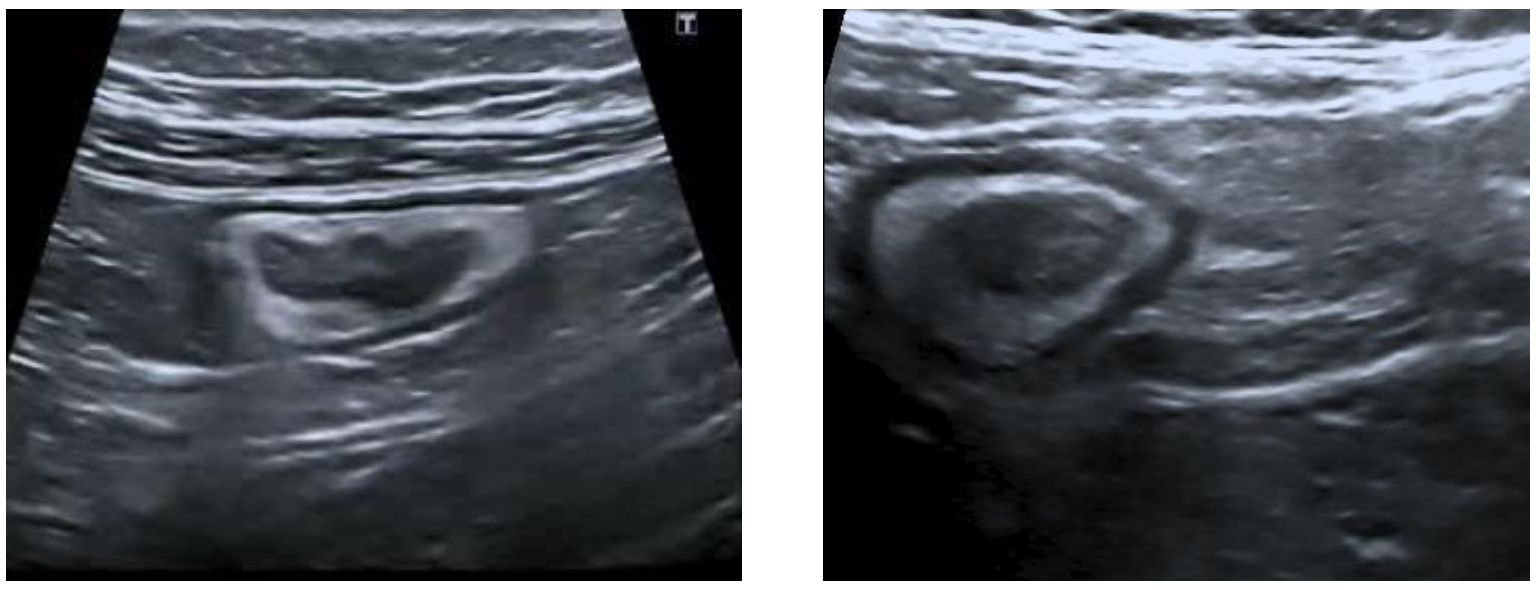

3 - Increased with bands (echogenicity greater than normal bowel in same patient with spoke wheel hypoechoic bands)

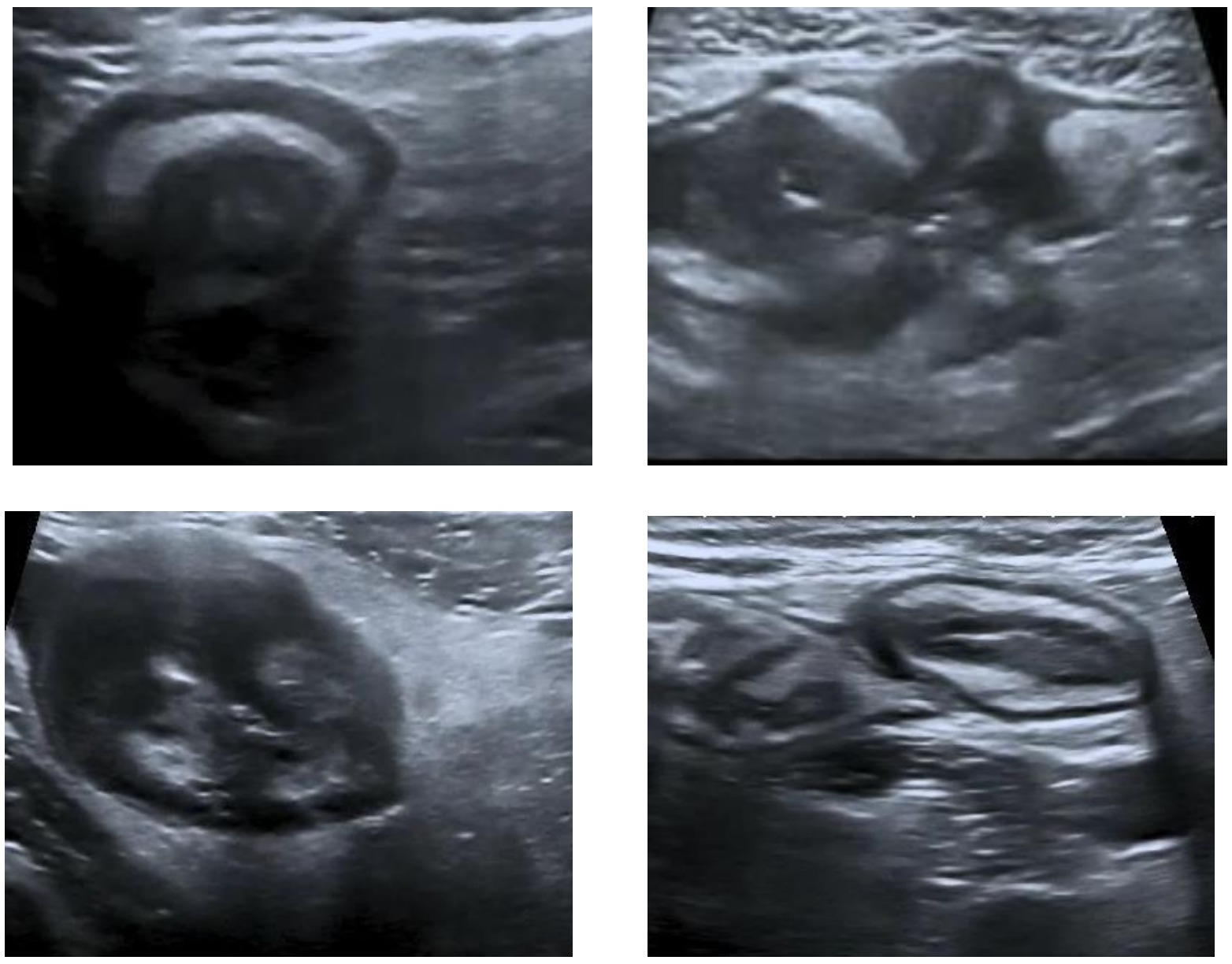

Case Report Form-USS SCORING

Sub-mucosal Layer Clarity

0 - Normal well defined (smooth interface between submucosa and other wall layers)

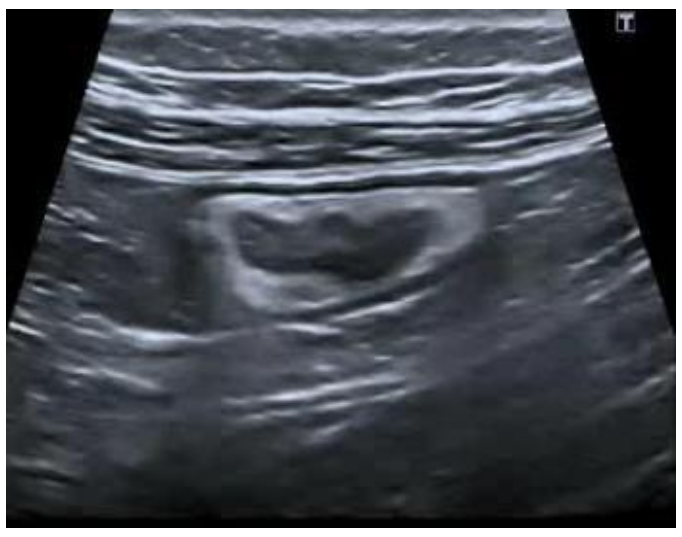

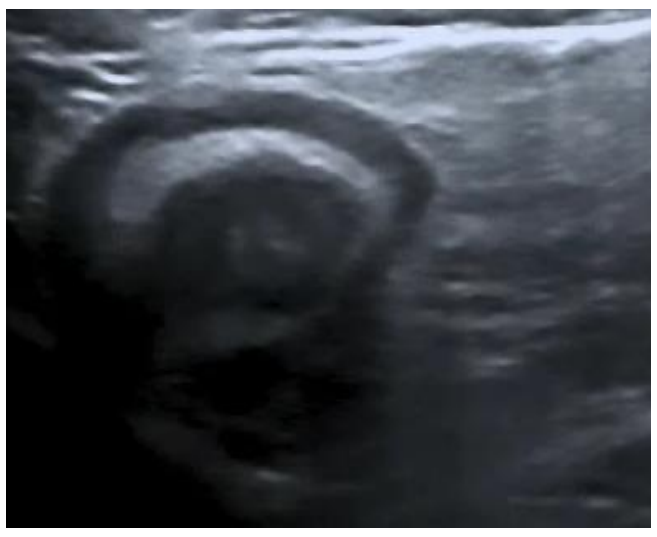

1 - Ill defined (irregular interface between submucosa and other wall layers)

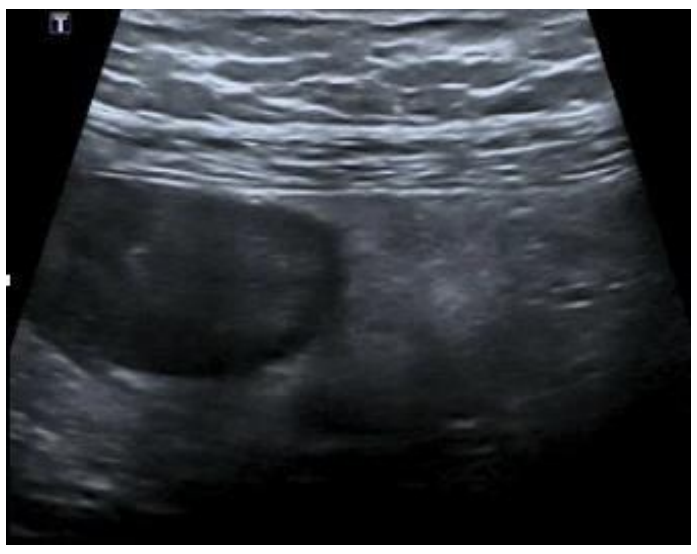

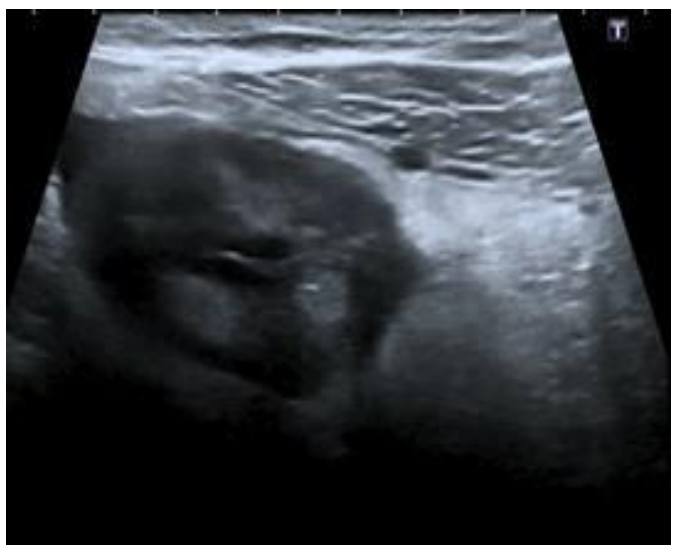

Mucosal layer

0 - Normal

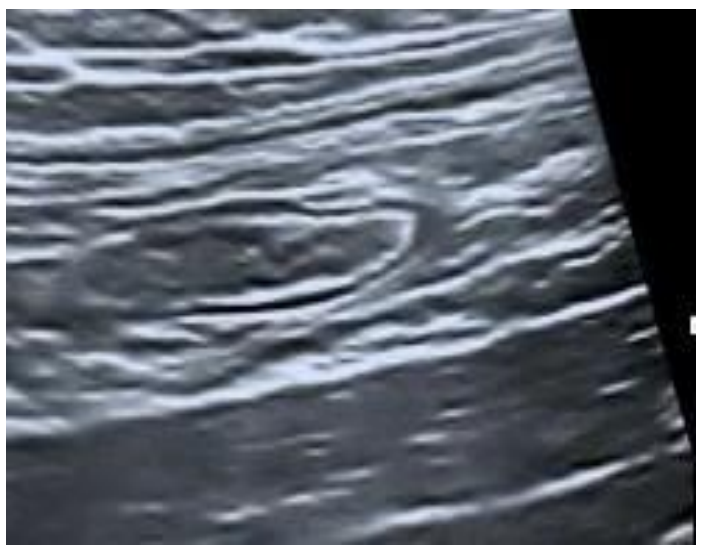

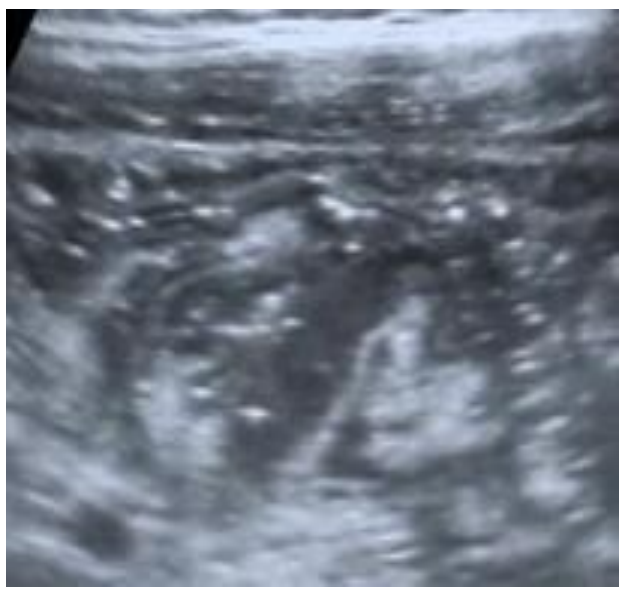

ileocaecal valve ulceration

1 - Isolated thickened (increased thickness compared to mucosa of normal bowel in the same patient

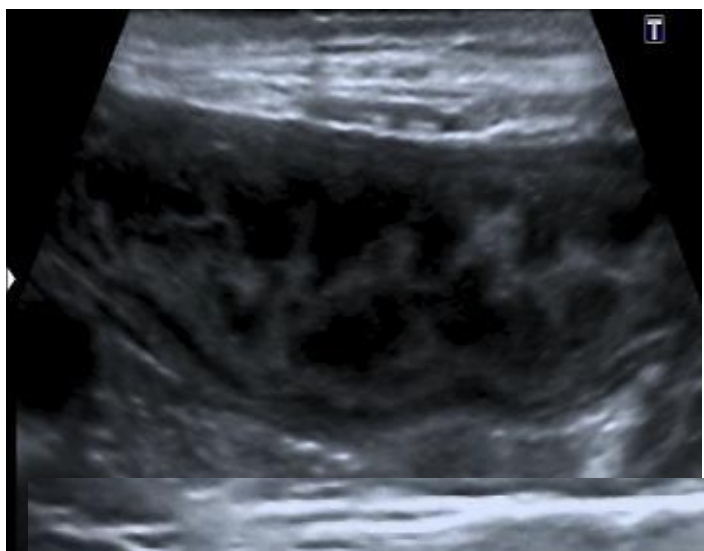

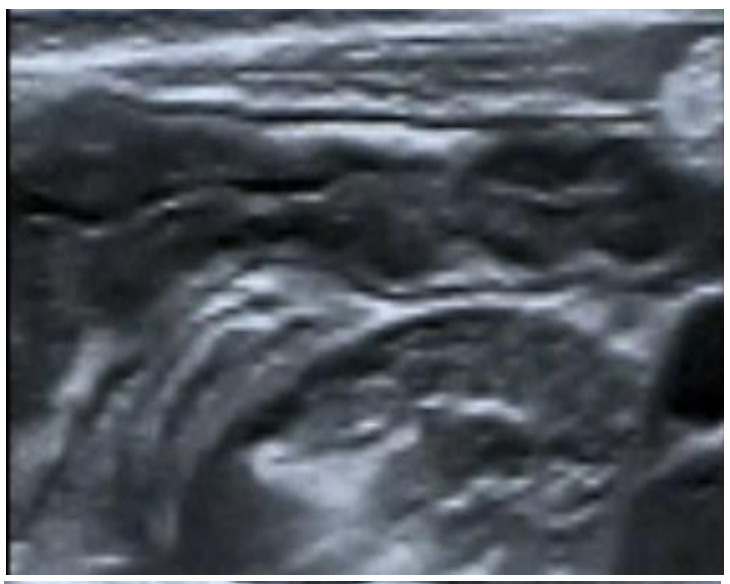

2 - Thickened also in the presence of submucosal thickening

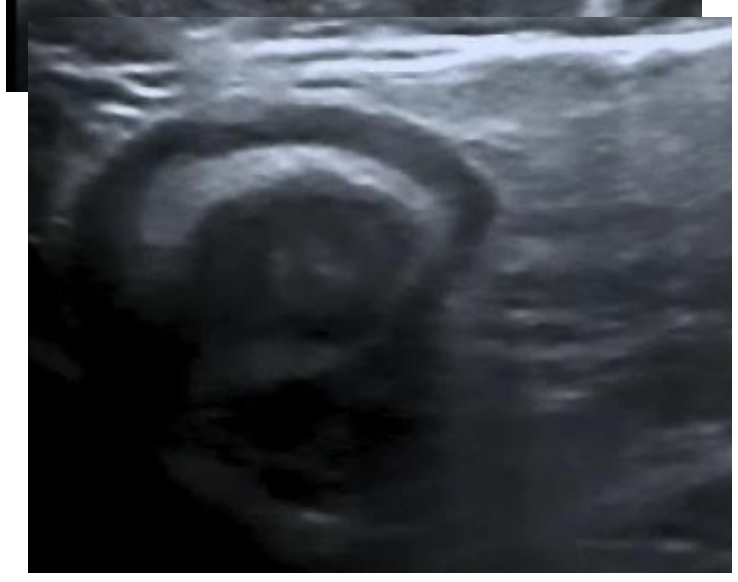

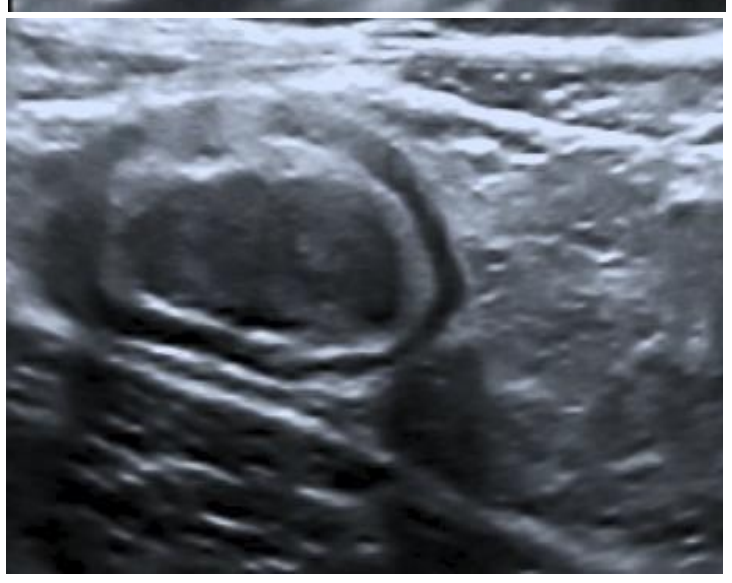

Case Report Form-USS SCORING

Doppler Vascular Pattern Imaging In Axial Section

0 - Normal

1 - Increased focal (increased Doppler signal isolated to less than half the circumference on a trans-axial image) compared to normal bowel in the same patient)

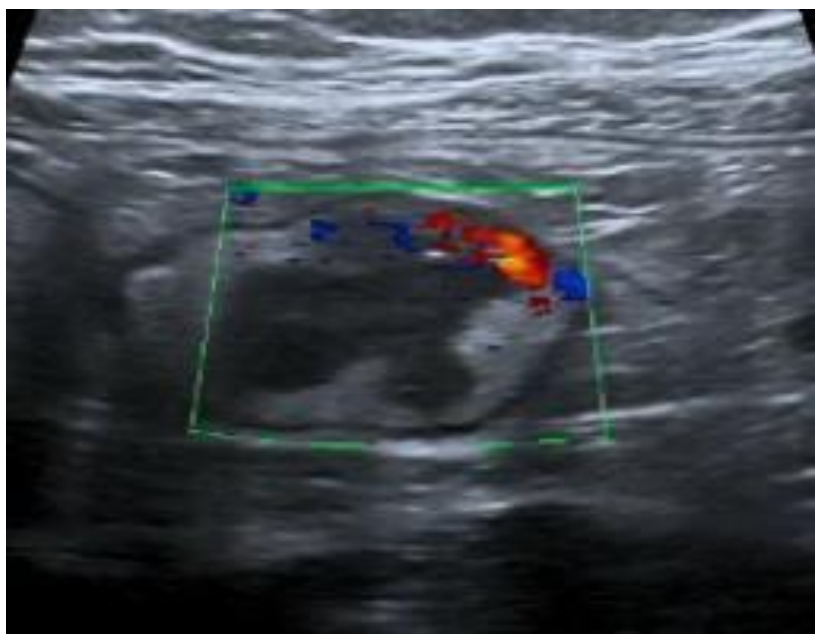

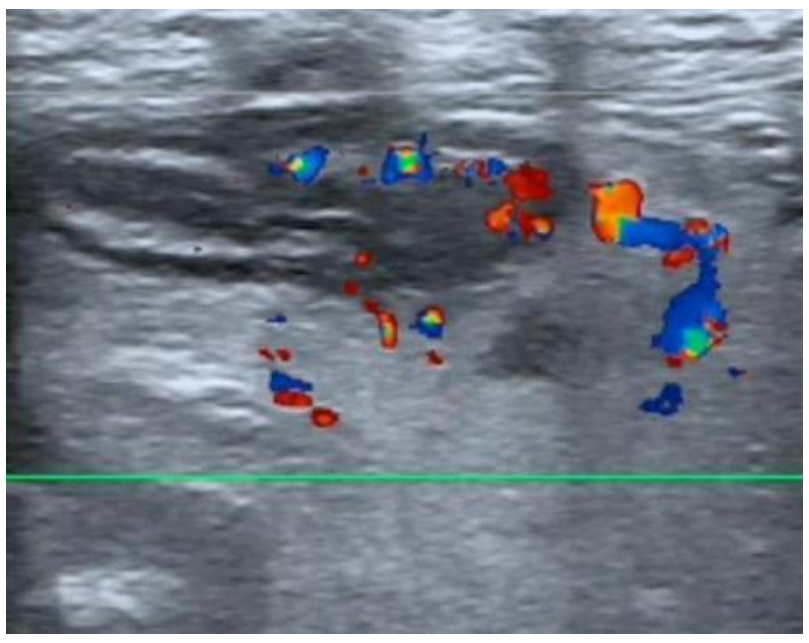

2 - Increased generalised (increased Doppler signal affecting more half the circumference on a trans- axial image compared to normal bowel in the same patient)

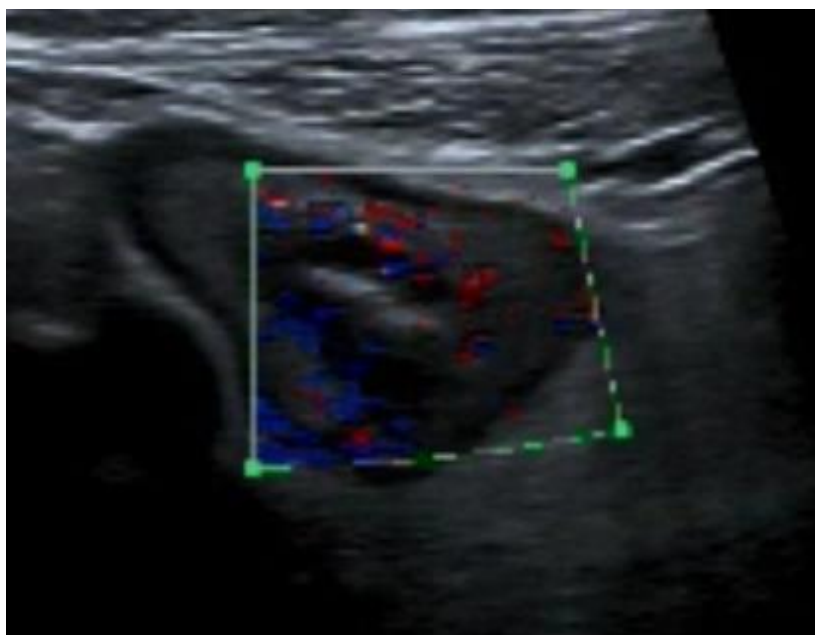

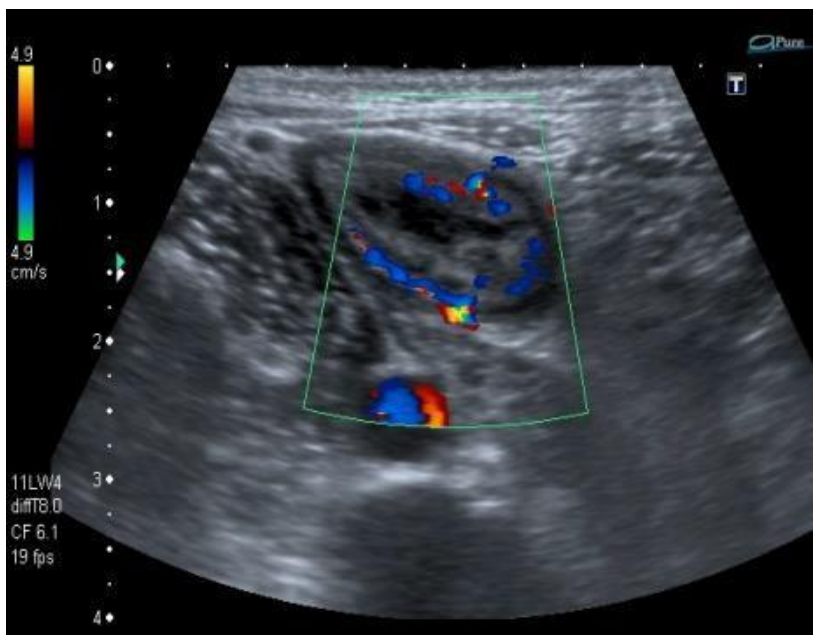

Ulceration

0- Normal

Superficial-< 50% wall thickness

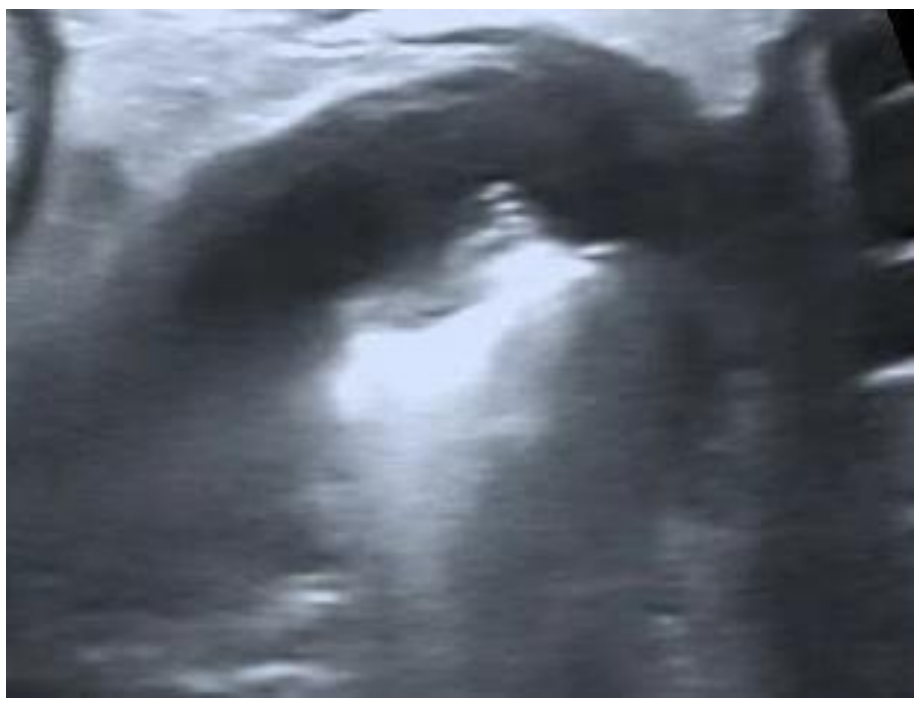

Deep-≥50% wall thickness

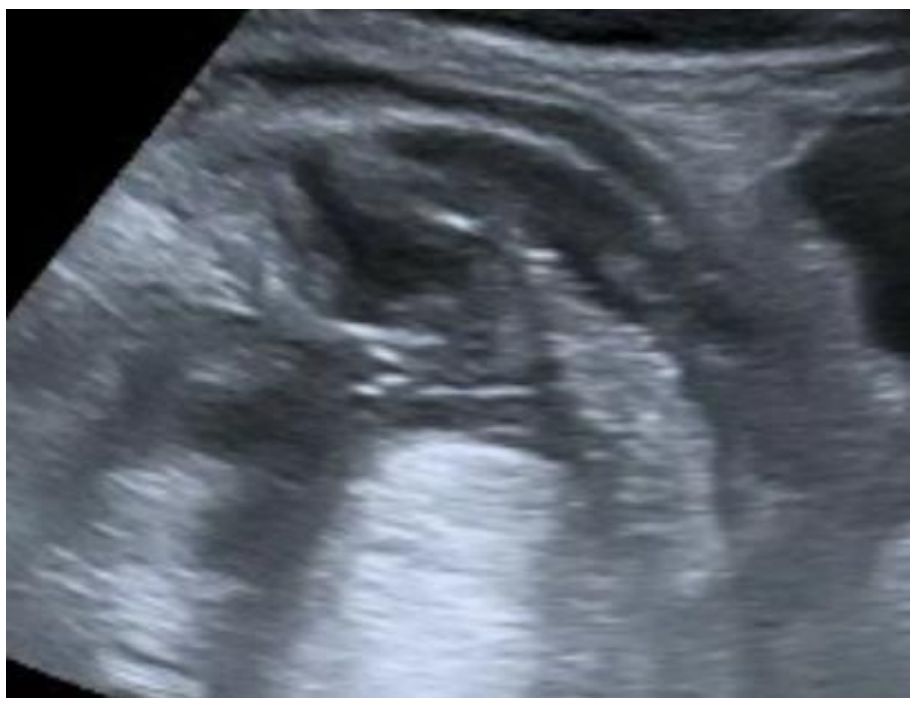

Case Report Form-USS SCORING

Functional obstruction = upstream dilatation

Peristalsis

1- Distension of the segment during peristaltic wave

2- Non distension of segment during proximal peristaltic wave

Disease activity At least one of: wall thickening/ focal hyperechoic mesentery (with or without fat wrap)/ isolated mucosal thickening /ill defined submucosal layer/ ill defined anti-mesenteric border/ Increased Doppler vascular pattern OR ulceration OR abscess

Disease assessment

Early-superficial ulceration and/or mild wall thickening/ mild increased vascularity Advanced-transmural disease and/or fistulation and/or stricturing and/or cobblestoning

/ and or wall oedema or significant thickening

Lymphadenopathy

0=none. 1=cluster all less than 1cm. 2=cluster 1 or2 nodes >1cm. 3=3 or more nodes greater than 1cm

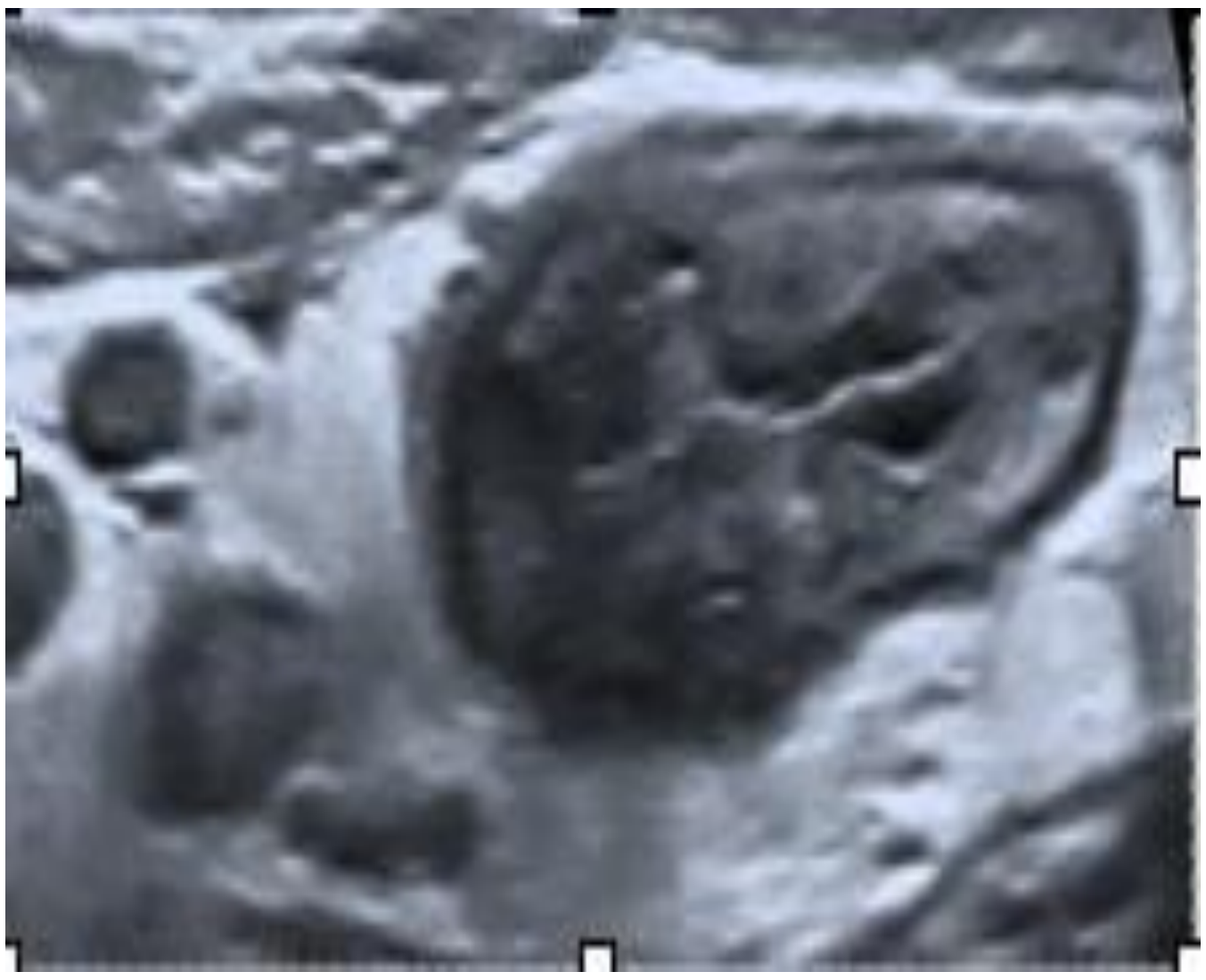

Appendix 2: Case report form for MRE and imaging key

PATIENT CASE REPORT FORM: MRI INTERPRETATION

Radiologist Initials_____________

MRI platform_____________________

Central review (due to unblinding at recruitment site) Y/N

Are you blinded to other clinical tests and investigations and patient clinical history (other than previous surgical history and new diagnosis or relapse cohort) Y/N

If N state what information/ test data you are aware of (e.g. barium FT)____________________

**Segment**	**Quality of segmental visualisation to make correct diagnosis**<br><br>**1-good/ 2-moderate/ 3-poor/ 4-N/A or excised**				**If poor visualisation, tick why**<br><br>**1- poor distension/ *2- oral contrast not reached segment/ 3-Other (state)***		
	1	2	3	4	1	2	3
**duodenum**							
**Jejunum***							
**ileum**							
**Terminal ileum****							
**Caecum**							
**Ascending**							
**Transverse**							
**Descending**							
**Sigmoid**							
**Rectum**							

* small bowel from DJ flexure mainly to the left of a diagonal running from the RUQ to LLQ showing typical feathery fold pattern, ** last 10cm of ileum upstream of IV valve/anastomosis

PATIENT CASE REPORT FORM: MRI INTERPRETATION

Overall disease assessment (to be completed for all patients)						
	Normal		Equivocal		Abnormal	
**Confidence of presence**	1 (disease definitely not present)	2 (disease probably not present)	3 (disease possibly not present)	4 (disease possibly present)	5 (disease probably present)	6 (disease definitely present)
**Any small bowel disease PRESENT?- tick confidence box**						
**Confidence of activity**	1 (disease definitely not active)	2 (disease probably not active)	3 (disease possibly not active)	4 (disease possibly active)	5 (disease probably active)	6 (disease definitely active)
**If present (confidence score ≥3) is it ACTIVE? tick confidence box**						
**Confidence of presence**	1 (disease definitely not present)	2 (disease probably not present)	3 (disease possibly not present)	4 (disease possibly present)	5 (disease probably present)	6 (disease definitely present)
**Any colonic disease PRESENT? tick confidence box**						
**Confidence of activity**	1 (disease definitely not active)	2 (disease probably not active)	3 (disease possibly not active)	4 (disease possibly active)	5 (disease probably active)	6 (disease definitely active)
**If present (confidence score ≥3) is it ACTIVE? tick confidence box**						

PATIENT CASE REPORT FORM: MRI INTERPRETATION

**Lymphadenopathy (0-3)**	☐ **0**	☐ **1**	☐ **2**	☐ **3**
**Abnormal free fluid (Y/N)**	☐ **Yes**		☐ **No**	
**Abscess present**	☐ **Yes**		☐ **No**	
If yes please state size & location				
**Fistula present (circle all that apply)**	☐ **Yes**		☐ **No**	
If yes please circle location		Ileo-ileal Ileo-colic entero-cutaneous ileo-vesical<br>colon-vesical jejuno- jejunal jejuno-colic<br>Other (state________________________________)		
**Other small bowel diagnosis (e.g. adhesions, meckels, radiation enteritis etc**	☐ **Yes**		☐ **No**	
**If yes please state**				
**Extra enteric findings (e.g. aortic aneurysm gallstones, solid organ abnormality, phelgmon)**	☐ **Yes**		☐ **No**	
If yes please state				
**Are you recommending any further tests?**	☐ **Yes**		☐ **No**	
If yes please state which				

PATIENT CASE REPORT FORM: MRI INTERPRETATION						
Please complete for each segment		**Confidence of disease PRESENCE**				
	**Normal**		**Equivocal**		**Abnormal**	
**Segment**	1 (disease definitely not present)	2 (disease probably not present)	3 (disease possibly not present)	4 (disease possibly present)	5 (disease probably present)	6 (disease definitely present)
**Duodenum (D)**						
**Jejunum (J)**						
**Ileum (I)**						
**Terminal ileum (TI)***						
**Caecum (C)**						
**Ascending colon (A)**						
**Transverse colon (T)**						
**Descending colon (D)**						
**Sigmoid (S)**						
**Rectum (R)**						

*throughout, if TI disease is contiguous for over 10cm count just as TI not TI and ileum

PATIENT CASE REPORT FORM: MRI INTERPRETATION

Confidence of disease ACTIVITY **Please complete for each segment if confidence scores 3-6 for disease presence above i.e. present or equivocal**

		Normal		Equivocal		Active	
**Segment**	No disease (i.e. confidence scores 1 or 2 for disease presence)	1 (disease definitely not active)	2 (disease probably not active)	3 (disease possibly not active)	4 (disease possibly active)	5 (disease probably active)	6 (disease definitely active)
**Duodenum (D)**							
**Jejunum (J)**							
**Ileum (I)**							
**Terminal ileum (TI)**							
**Caecum (C)**							
**Ascending colon (A)**							
**Transverse colon (T)**							
**Descending colon (D)**							
**Sigmoid (S)**							
**Rectum (R)**							

PATIENT CASE REPORT FORM: MRI INTERPRETATION Disease Description **Please complete for each disease site (defined as >3cm of normal bowel between disease sites). Use one table for each disease site. Only record segments which if you have a confidence score of 3 or more for disease presence.** ***Use score definitions at the start of this CRF***

DISEASE SITE 1

Location	Tick one location	Single Wall thickness (mm) thickest portion	Wall thickening	Length of abnormal bowel (cm)	Stensois causing functional obstruction	Peri-mural T2 signal	Mural T2 signal	Ulceration	Contrast enhancement	Contrast enhancement pattern	Diffusion signal	Does the segment contain established fibrosis (Y/N)	Segmental disease severity assessment	Segment shows active disease (Y/N)
Duo														
J														
I														
TI														
C														
A														
Des														
S														
R														

PATIENT CASE REPORT FORM: MRI INTERPRETATION Disease Description Please complete for each disease site (defined as >3cm of normal bowel between disease sites). Use <u>one</u> table for each disease site. Only record segments which if you have a confidence score of 3 or more for disease presence. *Use score definitions at the start of this CRF*

DISEASE SITE 2

Location	Tick one location	Single Wall thickness (mm) thickest portion	Wall thickening	Length of abnormal bowel (cm)	Stensois causing functional obstruction	Peri-mural T2 signal	Mural T2 signal	Ulceration	Contrast enhancement	Contrast enhancement pattern	Diffusion signal	Does the segment contain established fibrosis (Y/N)	Segmental disease severity assessment	Segment shows active disease (Y/N)
Duo														
J														
I														
TI														
C														
A														
Des														
S														
R														

PATIENT CASE REPORT FORM: MRI INTERPRETATION Disease Description Please complete for each disease site (defined as >3cm of normal bowel between disease sites). Use one table for each disease site. Only record segments which if you have a confidence score of 3 or more for disease presence. *Use score definitions at the start of this CRF*

DISEASE SITE 3

Location	Tick one location	Single Wall thickness (mm) thickest portion	Wall thickening	Length of abnormal bowel (cm)	Stensois causing functional obstruction	Peri-mural T2 signal	Mural T2 signal	Ulceration	Contrast enhancement	Contrast enhancement pattern	Diffusion signal	Does the segment contain established fibrosis (Y/N)	Segmental disease severity assessment	Segment shows active disease (Y/N)
Duo														
J														
I														
TI														
C														
A														
Des														
S														
R														

PATIENT CASE REPORT FORM: MRI INTERPRETATION Disease Description Please complete for each disease site (defined as >3cm of normal bowel between disease sites). Use one table for each disease site. Only record segments which if you have a confidence score of 3 or more for disease presence. *Use score definitions at the start of this CRF*

DISEASE SITE 4

Location	Tick one location	Single Wall thickness (mm) thickest portion	Wall thickening	Length of abnormal bowel (cm)	Stensois causing functional obstruction	Peri-mural T2 signal	Mural T2 signal	Ulceration	Contrast enhancement	Contrast enhancement pattern	Diffusion signal	Does the segment contain established fibrosis (Y/N)	Segmental disease severity assessment	Segment shows active disease (Y/N)
Duo														
J														
I														
TI														
C														
A														
Des														
S														
R														

PATIENT CASE REPORT FORM: MRI INTERPRETATION: Additional Sequences		
	Diffusion weighted	Contrast enhanced
Not helpful	☐	☐
Diagnosis unchanged but increased confidence	☐	☐
Diagnosis changed-additional disease site detected	☐	☐
Diagnosis changed-disease site now discounted	☐	☐
Diagnosis changed –disease re-classified as active	☐	☐
Diagnosis changed –disease re-classified as inactive	☐	☐
Other-state		

Completed by:
Print name: *Signature:* *Date:*

Office Use Only:
Received by (Print name & sign): *Date:* *Entered by: (Print name & sign):* *Date:*

MRI Imaging Key

Definitions of qualitative MRI scoring Index

Score	0	1	2	3
**Mural thickness**[a]	1-3mm	>3-5mm	>5-7mm	>7mm
**Mural T2 signal**[b]	Equivalent to normal bowel wall	Minor increase in signal – bowel wall appears dark grey on fat saturated images	Moderate increase in signal – bowel wall appears light grey on fat saturated images	Marked increase in signal – bowel wall contains areas of white high signal approaching that of luminal content
**Perimural T2 signal**	Equivalent to normal mesentery	Increase in mesenteric signal but no fluid	Small fluid rim (<2mm)	Large fluid rim (>2mm)
**Mural enhancement pattern**	N/A	Homogenous	Mucosal	Layered
**Enhancement**[b]	Equivalent to normal bowel wall	Minor enhancement – bowel wall signal greater than normal small bowel but significantly less than nearby vascular structures	Moderate enhancement – bowel wall signal increased but somewhat less than nearby vascular structures	Marked enhancement – Bowel wall signal approaches that of nearby vascular structures
**Lymph nodes**[d]	Absent	Cluster less than 1cm	1 node greater than 1cm	3 nodes greater than 1cm
**Lymph node enhancement**[c]	Less than nearby vascular structure	Equivalent or greater when compared to nearby vascular structures		
**Comb sign**	Absent	Present		

[a] Measured using electronic calipers, on HASTE sequences

[b] Compared to normal small bowel,

[c] Compared to nearest vessel

[d] Lymph nodes should be measured in short axis diameter

**Diffusion**	
**Score**	
**0**	Equivalent to normal bowel
**1**	minor increase in signal-bowel wall just visible on highest B value image
**2**	moderate increase in signal-bowel wall- easy to see on highest B value images
**3**	marked increase in signal- bowel wall very easy to see on highest B value images
**Ulceration**	
**Score**	
**0**	None
**1**	Superficial-< 50% wall thickness
**2**	Deep-≥50% wall thickness
**Functional obstruction = Upstream dilatation**	
**Score**	
**0**	None
**1**	Present
**Disease activity**	
**at least one of wall thickening/ increased mural signal/increased mesenteric signal / increased (enhancement mucosal or layered) OR ulceration OR abscess**	

**Segmental disease severity assessment score**	
**0**	None
**1**	Early-superficial ulceration and/or mild wall thickening/ mild increased vascularity
**2**	Advanced-transmural disease and/or fistulation and/or stricturing and/or cobblestoning/ and or wall oedema or significant thickening
